# Parasite Spillover from Domestic Sheep to Wild Reindeer—The Role of Salt Licks

**DOI:** 10.3390/pathogens12020186

**Published:** 2023-01-25

**Authors:** Kjersti Selstad Utaaker, Bjørnar Ytrehus, Marie L. Davey, Frode Fossøy, Rebecca K. Davidson, Andrea L. Miller, Per-Anders Robertsen, Olav Strand, Geir Rune Rauset

**Affiliations:** 1 Norwegian Institute for Nature Research (NINA), Torgarden, P.O. Box 5685, 7485 Trondheim, Norway; 2 Faculty of Bioscience and Aquaculture, Nord University, 8049 Bodø, Norway; 3 Department of Biomedical Science and Veterinary Public Health, Swedish University of Agricultural Sciences, P.O. Box 7028, 750 07 Uppsala, Sweden; 4 Norwegian Veterinary Institute, Holtvegen 66, 9016 Tromsø, Norway; 5 Department of Forestry and Wildlife Management, Faculty of Applied Ecology and Agricultural Sciences, Inland Norway University of Applied Sciences, 2480 Koppang, Norway

**Keywords:** attraction sites, disease transmission, spillover, domestic sheep (*Ovis aries*), reindeer (*Rangifer tarandus*), gastrointestinal nematodes, prion, chronic wasting disease

## Abstract

Attraction sites are important for environmental pathogen transmission and spillover. Yet, their role in wildlife disease dynamics is often poorly substantiated. Herein, we study the role of salt licks as potential attraction sites for the spillover of gastrointestinal parasites from domestic sheep to wild reindeer. Eggs from the introduced sheep nematode *Nematodirus battus* were found in faecal samples of both species, suggestive of spillover. DNA metabarcoding of soil, collected at salt licks, revealed that *N. battus*, in addition to *Teladorsagia circumcincta*, were the most frequently occurring parasitic nematodes, with a significantly higher prevalence of nematodal DNA in salt lick soil compared to soil from control sites nearby. The finding of similar DNA haplotypes of *N. battus* in sheep, reindeer, and salt lick soil supports the hypothesis of spillover to reindeer via salt licks. More detailed investigation of the genetic diversity of *N. battus* across these hosts is needed to draw firm conclusions. Infection with these sheep nematodes could potentially explain a recently observed decline in the calf recruitment rate of the Knutshø reindeer herd. This study also supports the hypothesized role of artificial salt licks as hot spots for the transmission of environmentally persistent pathogens and illustrates the importance of knowledge about such attraction points in the study of disease in free-roaming animals.

## 1. Introduction

The wild reindeer (*Rangifer tarandus tarandus*) population of Southern Norway is regarded as the last wild remnants of the tundra reindeer that once roamed large parts of post-glacial Europe. This leaves Norway with a specific responsibility for the conservation of European wild reindeer according to international conventions. Archaeological evidence indicates that the reindeer once migrated freely along the central mountain ranges of Southern Norway. Increasing human infrastructure and disturbance over the centuries has created obstacles that have now fragmented the population into 24 more or less isolated subpopulations. These subpopulations are under threat from multiple factors, including global warming with associated landscape changes and, most importantly, further human encroachment and disturbance. This leaves them vulnerable to further anthropogenic disturbances and stochastic events. Consequently, the population recently changed status from “Least Concern” to “Near Threatened” [1] on the Norwegian Red List of Species. Internationally, the species is listed as “Vulnerable” [2]. 

Wild reindeer populations are managed at a local level as separate units, in accordance with regional plans. Many of the larger populations are monitored by the National Monitoring Program for Wild Cervids [3] or are monitored by local stakeholders using similar methods. During the last few decades, many populations have seen declines in calf numbers and carcass weights. For example, the population in Knutshø used to have amongst the highest reproduction rates, highest calf weight gains, and highest carcass weights of adults in Norway. Over the last two decades, calf numbers and calf weights have declined. Stable carcass weights among yearlings and adults would suggest that poor pastures may not be the principal driver of these changes. 

**Figure 1:**
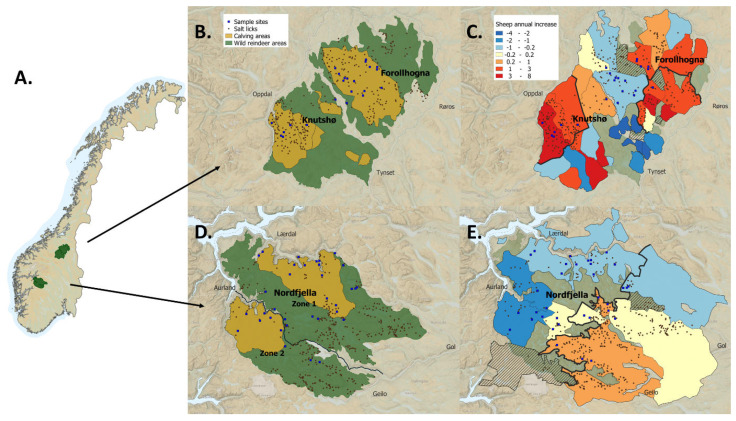
A: Map of the Nordfjella, Knutshø, and Forollhogna study areas with (**A**) their geographical location in Norway, (**B**,**D**) wild reindeer population areas (green = all year ranges, yellow = calving season, i.e., 1 May–20 June ranges) with recorded salt lick sites for sheep within the reindeer ranges and soil sample sites, and (**C**,**E**) annual change (percent) in total sheep numbers (ewes and lambs) in cooperative grazing districts during 1992–2018, based on linear regression models. The shaded areas represent grazing districts where we have incomplete data series. Borders between grazing subareas are represented with bold black lines. There are also salt lick sites outside the reindeer ranges. To improve clarity, these are not shown.

In 2016, we hypothesized that increased parasite load could be a contributing factor that could selectively affect calf survival and body mass. We started a pilot study to gain an overview of gastrointestinal parasites found in Knutshø and two comparable wild reindeer populations, Nordfjella and Forollhogna (Figure 1). Preliminary examinations of faecal samples collected from wild reindeer shot during ordinary autumn hunting revealed the presence of the introduced intestinal sheep parasite *Nematodirus battus* in some of the animals. This parasite was introduced to Norway from the UK in 1956 via imported sheep and typically causes enteritis and severe diarrhoea in pre-weaned lambs [4].

Sheep also frequently graze in the wild reindeer areas that were part of this study. Sheep husbandry in Norway is predominantly based on the utilization of upland grazing resources (natural and semi-natural pastures). Each summer, about 2 million sheep are released to range freely, most of them in alpine areas. Grazing is well organized, and most of the farms participate in cooperative grazing districts (“beitelag” or “sankelag”) that is economically supported by the authorities. While the number of other livestock has decreased in the last decades, the number of sheep has remained relatively stable, albeit with regional and local variations. The profit margin has, however, decreased whilst flock sizes have increased, with a decreasing proportion of the sheep farmers listing sheep production as their primary income and instead work off the farm [5]. Legislation demands that each farmer inspect all animals on outfield pastures on a weekly basis [6] so there is an incentive for the farmers to control the movements of the sheep at pastures to facilitate supervision. This is mainly done by placing salt stones at strategic, permanent locations. According to information from local managers and our own observations, an increasing number of permanent salt licks have been established in most of the mountain sheep grazing areas during the last two to three decades. To document and quantify this development has, however, been exceedingly difficult, as salt lick use and placement is not registered in any public registry. 

A number of factors therefore lead us to consider if parasite load could be an explanatory factor behind the decline in wild reindeer performance. A combination of increasing sheep flock sizes on upland pasture, the use of permanent salt licks in wild reindeer areas, and the finding of the sheep parasite *N. battus* in reindeer calves all hinted that spillover through exposure to sheep parasites at salt licks could be an explanatory factor behind the performance decline. We consequently hypothesized that *N. battus,* and possibly other parasitic nematodes, have spilled over from domestic sheep to reindeer through the common use of salt licks and have established a lifecycle in wild reindeer. 

In the current study, we used DNA metabarcoding of soil samples and examined parasite prevalence at salt lick sites frequented by both sheep and reindeer, as well as at comparable control sites. In addition, we examined nematode DNA haplotypes found in soil samples, domestic sheep, and wild reindeer in the Knutshø, Nordfjella, and Forollhogna areas to corroborate or refute the spillover hypothesis. 

## 2. Materials and Methods

### 2.1. Ethical Statement

The current study does not involve experiments or handling of humans or animals but only observational studies of natural and undisturbed behaviour and collection of samples from animals shot during ordinary hunting, and is hence not required to apply for ethical approval according to Norwegian legislation or institutional policy.

### 2.2. Study Areas

In the current study, we focused on three wild reindeer management areas: Nordfjella, Knutshø, and Forollhogna, where the latter two have been included in the National Monitoring Program for Wild Cervids since 1991. 

Nordfjella is a 3000 km^2^ area of rugged mountains, between the marine fjords of Western Norway and the valley landscape of the eastern part of the country (60°50′ N, 7°44′ E) [7]. Much of the area is in the upper alpine vegetation zone, but with intersecting valleys with lush pasture. Human infrastructure (hydroelectric dams, cabins etc.) has reduced the available area for the reindeer, and the area is intersected by a road (FV 50), dividing the reindeer subpopulation in two functional zones. A herd of about 2000 animals in the northern zone was eradicated in 2018 in order to minimize the risk of spreading Chronic Wasting Disease (CWD), after its discovery in 2016, while a herd of about 500 reindeer persisted through the study period in the southern zone. During the two decades before the eradication program, the population size was stable, but some data indicated that the reindeer aggregated in the areas less disturbed by humans, and that lichen pastures in these areas were reduced. During the monitoring period of 1991–2018, the reindeer calf recruitment rate varied, with a slight negative trend (see result section).

Knutshø is a 1776 km^2^ area of rounded mountains in Central Norway (62°25′ N, 10°0′ E) [7]. The area has rich mountain pastures in the medium and lower alpine vegetation zones, but is highly influenced by human encroachment with hydroelectric dams and construction roads, also increasing accessibility for humans and thereby anthropogenic disturbance. Consequently, the reindeer population of 1500–1900 animals utilizes only a small part of the pastures that, in theory, should be available to them. Three decades ago, the population was regarded as one of the most productive wild reindeer herds in Norway. Since 2000, both the number of calves per female and the body condition of the calves shot during hunting have declined.

Forollhogna is a 1843 km^2^ area of gently rounded mountains north of Knutshø (62°40′ N, 10°45′ E) [7]. The mountains are interspersed with dairy farm valleys, with rich pasture in the medium and lower alpine vegetation zones. There are hydroelectric dams and construction roads in some parts of the area, but less human disturbance than in Nordfjella and Knutshø. Forollhogna is also the wild reindeer management area closest to the area where CWD was first discovered in moose [8]. The reindeer herd of 2000–2200 individuals have the highest performance of all Norwegian wild reindeer herds, but since 1991, the calf recruitment rate also had a slight negative trend (see Section 3). 

### 2.3. Sheep Production and Area Use

**Figure 2:**
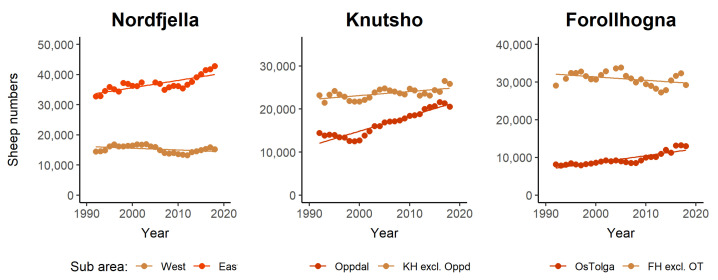
Total sheep numbers (ewes and lambs) of organized grazing units in Nordfjella, Knutshø, and Forollhogna during 1992–2018, each divided into two subareas. Trend lines are predictions from linear regression models.

Data about sheep production in Norway was retrieved from Statistics Norway [5] and a database on subsidies driven by the Norwegian Agricultural Directorate (www.data.norge.no, accessed on 18 March 2020). Data on the use of the grazing districts in Nordfjella, Knutshø, and Forollhogna were retrieved from the database “Organisert beitebruk” with the help of Michael Angeloff at the Norwegian Institute for Bioeconomics (NIBIO) on the 18 March 2020, while shapefiles of grazing district borders and other geographical information were collected from https://kilden.nibio.no (accessed on 29 September 2022). Supplemental data on numbers of sheep in Folldal, Rennebu, and Oppdal municipalities were retrieved from public reports on municipal web pages [9,10,11]. Linear regression models were used to analyse the development in total sheep numbers (ewes and lambs) within individual grazing districts during 1992–2018. All statistical analyses were performed in Program R and RStudio (R Core Team 2022, RStudio Team 2022). Only districts with a time series spanning > 15 years were included in these analyses. Spatial variation in sheep number development was plotted using QGIS v. 3.16.4 (Figure 1C,E). To analyse major patterns of within-area trends in sheep numbers, each study area was divided in two subareas, identifying the parts of the area that constituted the bulk of increasing herd sizes. Thus, Nordfjella was split into a western (Lærdal, Austfjelli, Vestfjelli, and Stemmerdalen) and an eastern part (Iungsdalen, Fødalsdrifta, Hemsedal, Ål, and Hol). Knutshø was divided by grazing districts within Oppdal municipality vs. all the remaining areas. Forollhogna was divided by grazing districts within Tynset and Tolga municipalities vs. all the remaining areas. Differential trends in sheep numbers among these subareas during 1992–2018 was analysed using linear regression models (Figure 2).

### 2.4. Reindeer Population Monitoring and Area Use

Eight of the largest wild reindeer populations are surveyed annually as a part of the National Monitoring Program for Wild Cervids. The surveys are organized by The Norwegian Institute for Nature Research (NINA) in cooperation with the local management boards. Data include: (a) aerial counts of “nursing herds” (consisting of mainly adult females with calves and subadults, during the summer to assess calf recruitment; (b) harvest data reported by hunters (hunting season, 20 August–30 September); (c) on-ground counts during the fall rutting season (when all demographic groups aggregate, late September–October) to assess population structure; and (d) aerial counts during winter to assess the total population size (“minimum count”) [12]. In addition, a few additional populations are monitored locally using similar techniques. The numbers represent population level data with no local variation. All population monitoring data are available from the Norwegian Environment Agency (https://www.hjorteviltregisteret.no (accessed on 29 September 2022)) in Norwegian). 

**Figure 3:**
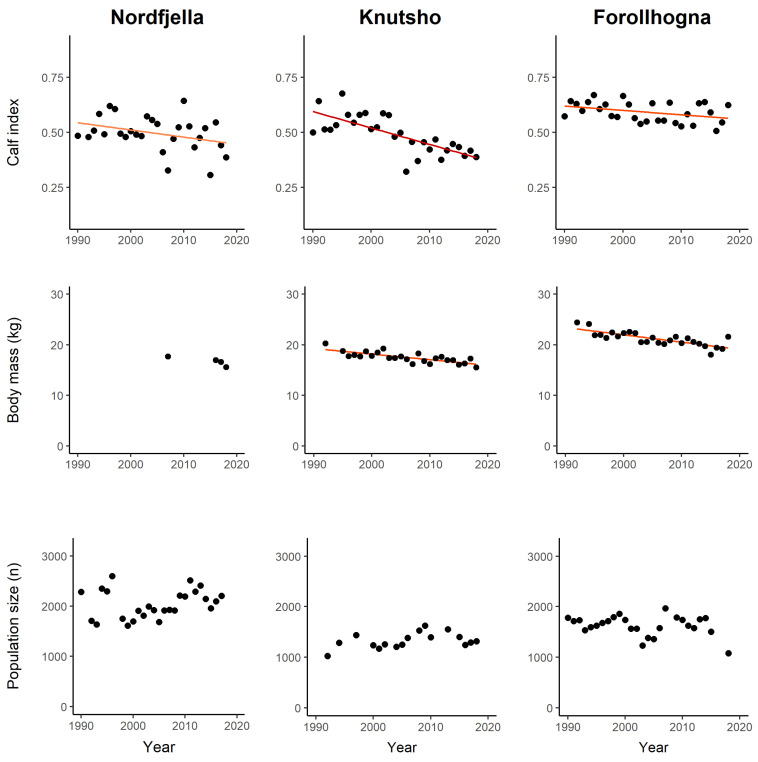
Wild reindeer calf recruitment rates (top row), body mass (middle row), and population size (bottom row) in Nordfjella, Knutshø, and Forollhogna study areas during 1990–2018. Calf recruitment is defined as the number of observed calves per adult female and subadults during summer aerial counts. The calf body mass is the mean dressed weight of harvested calves (standardized to a female individual shot on the 4 September). The population size is the total number of observed reindeer from winter aerial counts. Trend lines (orange to red) are predictions from linear regression models. Non-significant trends have no trend line.

To investigate trends in calf recruitment for the focal reindeer populations, we used summer aerial count data collected by NINA (Knutshø and Forollhogna) and Aurland Fjellstyre (Nordfjella) from 1990 to 2018. For each population, the mean number of observed calves per adult and subadult reindeer was analysed as a time series with general linear models and plotted in Figure 3 (top row). To investigate trends in calf body mass, we used data on the dressed weight of harvested calf carcasses from 1990 to 2018. We calculated the median date for all calves harvested (4 September). We fitted general linear models with year, sex, and Julian date as explanatory variables, and plotted model predictions of the mean annual dressed weight, standardized to a female individual harvested on the 4 September (Figure 3, bottom row). 

Wild reindeer area use in a representative sample of population (including the three study populations) was recently reviewed by NINA and the Norwegian Wild Reindeer Center in cooperation with local management boards [7]. Data included (a) all year ranges, (b) calving season (1 May–20 June) ranges (c) winter ranges, and (d) migration routes. This data are available from the Norwegian Environment Agency, and maps on seasonal range use were downloaded as shapefiles from their map database (https://www.miljodirektoratet.no/tjenester/naturbase/, accessed on 29 September 2022).

### 2.5. Collection and Parasitological Examination of Faecal Samples

Faecal samples were collected by reindeer hunters in 2016 during the annual autumn wild reindeer hunt in Forollhogna, Knutshø, and Nordfjella. The collection was continued in Knutshø in 2017, but not in Nordfjella and Forollhogna. Hunters were supplied with sample kits (gloves, sample containers, instructions for sampling, and a pre-postage paid envelope addressed to the laboratory). The hunters were asked to use a glove, collect the faeces directly from the rectum, and put it in the plastic container to avoid soil contamination. They were instructed to mark the container with the animal category (calf, yearling, adult female, or adult male), tag number supplied with the hunting permit card, and the date the animal was shot, and then, submit the sample as soon as possible. In Forollhogna, the sample collection was performed by local mountain rangers. Transport from the field to the laboratory was 12 days on average in 2016 (median 9, maximum 56, and minimum 2 days), and in 2017, the transportation time was 8 days on average (median 7, maximum 15, and minimum 3 days). Upon arrival at the laboratory, the samples were stored at 4 °C for a maximum of 3 days before processing. Faecal samples from the same areas were also collected during the ordinary hunting season in 2018 and 2019 in relation to another still ongoing project. Here, the sampling from all three areas was done in collaboration with mountain rangers and hunters, who provided the position of the offal as well as the age category and sex of the animal. The offal was located using GPS location, and the samples were retrieved. The samples were transported to the laboratory within 72 h after the collection from the field and stored at 4 °C until processing (within 1 week). The sampling in Nordfjella was more complicated logistically and resulted in a longer time (up to 3 weeks) between field sampling and proper laboratory storage. The results from analysis of these samples are also presented in this study (See result section)).

Faecal samples from sheep grazing in Nordfjella zone 2, Forollhogna, and Knutshø were collected in collaboration with a farmer from each of the three areas. Samples were collected at the respective local slaughterhouses. All sheep samples were collected in autumn 2018 and 2019, and the sheep had been sent to slaughter no more than 2 weeks after return from natural pastures. 

The intensity of endoparasite eggs and oocysts was estimated using a modified McMasters technique and zinc–chloride/sodium chloride flotation fluid (with a specific gravity of 1.3) [13]. A 3-gram sample was homogenized in 57 mL water and sieved through a sieve with a pore diameter of 250 µm or ≈1000 µm. The suspension was divided into two 15 mL tubes and centrifuged at 1550 relative centrifugal force (rcf/G) for 3 min, after which the supernatant was discarded. The concentrated pellet from one of the tubes was re-suspended in the flotation fluid. The McMaster counting chambers (Chalex LCC or Whitlock Universal, Granwille, NSW, Australia) were filled with 2 or 2.5 mL of this suspension, and the whole slide was read at 100–200× microscope magnification for the detection and quantification of parasite eggs. Parasite eggs and oocysts were identified to order (Strongylida), subfamily (Nematodirinae), genus (*Eimeria* sp., *Moniezia* sp., and *Trichuris* sp.), and species levels (*Nematodirus battus* and *Strongyloides papillosus*) based on their morphological characteristics. 

### 2.6. Isolation of Nematodirus battus Eggs from Reindeer Faeces 

*N. battus* eggs were isolated from wild reindeer faecal samples collected from calves in 2019. The samples contained 70, 150, and 310 *N. battus* eggs per gram. An in-lab protocol used for the isolation of *Eimeria* sp. oocysts was applied, with a few modifications, to isolate *N. battus* eggs. In brief, faeces were washed in several steps followed by flotation using NaCl/ZnCl_2_ solution (specific gravity 1.36× *g*). The supernatant from the flotation procedure was washed with tap water and kept in petri dishes. Using a stereomicroscope and a 10 µL pipette, *N. battus* eggs were selectively transferred to a second petri dish containing tap water and stored at 4 °C.

### 2.7. Molecular Biology

To examine the genetic diversity of *N. battus* isolated from sheep and reindeer, we retrieved the ITS sequences from NCBI non-redundant nucleotide databases, including 8 *N. battus* worms isolated from sheep and reindeer in the focal areas as described by Davey et al. [14] (Table 1). Percentage identity was compared pairwise between the sequences using the *pid* function in the *Biostrings* package in R [15], and a sequence alignment of these and the 11 most frequent *N. battus* amplicon sequence variants generated from amplicon sequencing of soil samples. These reference sequences and amplicon sequence variants were aligned with an additional 17 *N. battus* sequences retrieved from GenBank and a multiple sequence alignment was generated in Geneious 2020. The alignment was subsequently plotted with the *ggmsa* package in R [16] and linked to host and geographic provenance information for each sequence.

### 2.8. Soil Sample Collection and Examination 

Localities were selected based on local knowledge regarding the use of salt lick sites by reindeer and other wild cervids. We tried to select soil samples from salt licks representing the variation within each mountain area (altitude, longitude, topography, and vegetation). For this study, a total of 178 soil samples were collected in the summer (June-July) and/or fall (August–October) field campaigns during 2017–2019. Forty-four localities currently in use, or formerly used, as salt licks were sampled from 1 to 5 times during the study period along with soils from nearby control localities that showed no signs of animal congregation or use (Table S1). In total, 10 salt licks were sampled in Forollhogna, 7 in Knutshø, and 25 in Nordfjella. One abandoned salt lick (currently without a salt lick) was sampled in Forollhogna, and five abandoned salt licks were sampled in Nordfjella, all of which occurred in close proximity to active salt licks (Table S1). Parallel to this, we monitored the use of these salt lick sites by sheep and wildlife, using motion triggered camera traps (Recognyx XP9 UltraFire and Browning Strike Force HD). Soil samples from selected salt licks were collected according to the following procedure: A two-meter ruler was laid on the ground with one end against the pole holding the salt stone and positioned so that it followed the main direction of water flow. Two other rulers were placed in a 45° angle to this first ruler so that they were at 90° angles to each other with the first in the middle. Loose faeces, dirt, and debris were removed from the soil surface before the upper 2–5 cm of soil was sampled using a tablespoon. A spoon of soil was collected every 20 cm along the rulers, starting 20 cm from the salt lick, and stopping at the 2-m mark. If stones or solid rock prevented sampling in a sampling point, additional sampling points were made every 20 cm in the direction of the ruler until 10 samples were taken along each. The resulting 30 subsamples were gathered in a cylindrical plastic container, making up approximately 500 mL of soil. The container was closed with an airtight lid and thoroughly shaken to mix the spoonfulls of the sample. A control site was chosen 300–500 m from each sampled salt lick. The control sites lay on the same altitude and had similar topography, slope, and vegetation as the salt lick, but without visible signs of frequent animal use. 

The samples were frozen at −12 °C before transport and during storage, and processed after thawing at 4 °C. The soil was sifted through a 2 mm sieve to remove larger objects such as stones and twigs, and thoroughly mixed before further processing. 

Approximately 15 mL of sieved soil from each sample was used as the starting material for DNA isolation using the 50 mL FastDNA™ Spin Kit for Soil (MP Biomedicals, San Diego, CA, USA). DNA was isolated according to the manufacturer’s protocol with the following modification: instead of using GTC wash buffer, columns were washed twice with SEWS-M buffer. The primers NC1 and NC2 [17] were used to amplify the ITS2 region of ribosomal DNA (rDNA) of Clade V parasitic nematodes. PCR reactions were conducted in 25 µL volumes containing 1× KAPA HiFi HotStart ReadyMix (Roche, Switzerland), 0.5 µM of the forward and reverse primers, and 50 ng of the template DNA. PCR-conditions consisted of an initial denaturing step of 5 min at 95 °C, followed by 35 cycles of 1 min at 95 °C, 1 min at 54 °C, and 1 min at 72 °C with a final elongation step of 5 min at 72 °C. Magnetic beads (MAG-BIND RXN PURE PLUS) were then used to clean PCR amplicons, removing fragments <200 bp and >600 bp, and indices were attached to the purified amplicons using the Nextera XT Index kit (Illumina, San Diego, CA, USA). Indexing reactions were conducted in 50 µL volumes containing 1x KAPA HiFi HotStart ReadyMix (Roche), 1 uM of the forward and reverse indexing primers, and 5 uL of template DNA. PCR conditions for indexing consisted of an initial denaturing step of 3 min at 95 °C followed by eight cycles of 30 s at 95 °C, 30 s at 55 °C, and 30 s at 72 °C, and a final elongation step of 5 min at 72 °C. Magnetic beads (MAG-BIND RXN PURE PLUS) were again used to clean the indexed amplicons, removing fragments <200 bp and >600 bp. Amplicons were then pooled in equimolar amounts and sequenced in a paired end 300 bp run on the Illumina Miseq sequencing platform at the Genomics Core Facility (GCF), Norwegian University of Science and Technology (NTNU), Trondheim, Norway.

The MiSeq platform was used to demultiplex samples, and cutadapt v 1.18 [18] was further used to remove the forward and reverse primers from each read, requiring a minimum match length of 17 bp, no indels, and <0.15 expected errors over the primer length. The DADA2 package in R [19] was used to conduct further quality filtering, error correcting, merging and chimera checking of the sequences. All sequences that were <50 bp, contained ambiguous bases, or >2 expected errors across the entire length of the forward read or >4 expected errors across the entire length of the reverse read were removed from the dataset, and sequences were truncated at the first instance of a base with Phred quality score <15. Forward and reverse reads were merged with a minimum overlap of 30 bp, and the *de novo* consensus method in DADA2 was used to remove chimeric sequences. More specifically, chimeras were assessed on a per-sample basis. Sequence variants that were flagged as chimeric, in more than 90% of the samples they occurred in, were removed from the dataset. Taxonomy was assigned to ASVs using the naïve Bayesian classifier [20] implemented in DADA2 and a custom version of the Nematode ITS2 v.1.0.0 database [21,22,23] including an additional six reference sequences from adult nematodes identified from reindeer hosts in an earlier study [14] and requiring a minimum confidence estimate of 80 percent to successfully match at any given taxonomic level. Each ASV was also subjected to a BLAST search against the NCBI nucleotide non-redundant database. Any ASV with a best BLAST match to a lineage outside the phylum Nematoda, or that could not be assigned with confidence >80% at the phylum level was designated a non-target amplification and excluded from further analyses. 

### 2.9. Statistical Analyses

A binomial generalized mixed model was used to investigate differences in parasitic nematode frequency between soils collected at active salt lick and control sites across the three focal areas. For each management area, the available species pool was considered to be the total number of unique species recovered from the area by DNA metabarcoding, across all localities during the study period. For each soil sample, parasitic nematode detection was then expressed as the cumulative number of detection successes and failures for the members of the local species pool, which was then used as the response variable for the model, and a random contribution from locality was included. We compared a null model with models including management area and salt lick status as fixed effects, as well as a combined model including their additive effects (Table S2). Results from the best fitted model based on Akaike Information Criterion (AIC) are presented. Models were run using the *lme4* package in R [24], and post-hoc odds ratios were calculated using the *emmeans* package [25].

Due to low numbers and an unbalanced sampling design (Table S1), a general linear model with no random contributions was used to investigate differences in parasitic nematode frequency between soils collected from control sites and salt lick sites that had been abandoned or were in active use at 5 localities in Nordfjella and 1 locality in Forollhogna. The model was run using the *stats* package in R [26] and included salt lick status (abandoned, active, control) as a fixed effect. Post-hoc odds ratios were calculated using the *emmeans* package.

The results from the McMaster analysis were recorded in a Microsoft Excel^®^ database. The same programme was used to compare the results with a chi-square test, and the Kruskal–Wallis test was performed using an online calculator [27].

## 3. Results

### 3.1. Sheep Production and Pasture Use

In Nordfjella, sheep production has been relatively stable, but, from 2000 to 2018, increased by about 10%. This increase occurred in the eastern parts of the area, while the number of sheep and lambs decreased slightly in the western grazing districts (Figure 2 and Figure 3). In 2018, about 62,000 sheep and lambs were released to graze in Nordfjella. In Knutshø, the number of sheep and lambs at pasture has increased by 27%. This is equivalent to an increase of approximately 10,000 additional animals between 2000 and 2018, and reached a total of 46,462 grazing sheep in 2018. Most of the increase occurred in the northwestern grazing districts in Oppdal municipality, i.e., Driva, Ålma-Gissingbekk, and Innsetmerket-Ålma (Figure 1). Here, the number of sheep and lambs increased from 12,752 in 2000 to 20,544 in 2018 (61%) (Figure 2). In Forollhogna, only a moderate overall increase (8%) was observed between 2000 and 2018, and about 40,000 sheep and lambs were released into the mountains. The numbers actually decreased in most of the areas, while there were increases in the northernmost and southernmost grazing districts (Figure 1 and Figure 3)

### 3.2. Reindeer Calf Recruitment and Body Mass

In Nordfjella, the number of calves and adult female/subadults observed during aerial counts in summer was 123–796 (mean = 477) and 318–1391 (mean = 943), respectively. The calf recruitment index (calf per adult female/subadult) tended towards a negative trend during 1990–2018 (t = −1.87, *p* = 0.072), with a 16.7% reduction based on model predictions. Data on calf body mass were only sporadic, with 25–87 calves reported in the years 2007, 2016, and 2017. Data were not judged sufficient for further analyses. The population size (i.e., minimum count during winter) in Nordfjella ranged between ca 1.600 and 2.600 individuals, with a mean value of ca 2.000, during 1990–2017. During the fall of 2017 and winter of 2018, the entire subpopulation of zone 1 (>2000 reindeer) was eradicated due to the local CWD outbreak, and year 2018 was not included in the trend analysis. Though there was substantial fluctuation in the population size over the period, there was no linear trend in the population size (t = 1.18, *p* = 0.25).

In Knutshø, the number of observed calves and adult female/subadults was 139–475 (mean = 351) and 419–1124 (mean = 742), respectively. The calf recruitment index (calf per adult female/subadult) had a highly negative trend (t = −5.69, *p* < 0.001), representing 35.1% reduction in calf recruitment during 1990–2018 based on model predictions. The annual number of the reported calf body mass was 22–77 (mean = 49). In a linear model with “Sex”, “Julian date”, and “Year” (continuous variable), the year parameter estimate was highly significant (t = −7.6, *p* < 0.001), representing 13.8% reduction of the calf body mass during 1992–2018. The Knutshø population size ranged between ca 1.000 and 1.600 individuals (mean ca. 1300). Despite some fluctuations in population size, there was no linear trend over the time period (t = 1.56, *p* = 0.13).

In Forollhogna, the number of observed calves and adult female/subadults was 253–805 (mean = 537) and 397–1413 (mean = 911), respectively. The calf recruitment index (calf per adult female/subadult) had a negative trend (t = −2.1, *p* = 0.048), representing 8.9% reduction in calf recruitment during 1992–2018 based on model predictions. The data on calf body mass was 78–318 (mean = 190) calves, reported annually during 1992–2018. In a linear model with “Sex”, “Julian date”, and “Year” (continuous variable), the year parameter estimate was highly significant (t = −25.1, *p* < 0.001), representing 21.5% reduction of calf body mass during 1992–2018. The Forollhogna population size ranged between ca 1.100 and 2.000 individuals (mean ca. 1600). The population size dipped two times during the period, including the year 2017–2018, which could, at both times, be attributed to human harvesting. Despite these fluctuations, there was no linear trend in population size over the time period (t = 1.14, *p* = 0.26).

### 3.3. Collection of Faecal Samples

In 2016 and 2017, 235 faecal samples were collected during the annual autumn wild reindeer hunt in Forollhogna (n = 84), Knutshø (n = 104), and Nordfjella (n = 47). In addition, 18, 22, and 8 samples were collected from fresh offal from hunted reindeer shot in 2018 and 2019 in Forollhogna, Knutshø, and Nordfjella, respectively. Among the sampled animals were 79 calves and 149 adults, while 55 samples were received without having the age or sex of animal specified. The results on prevalence and intensities of parasite eggs and oocysts in faecal samples from wild reindeer are presented in Table 2 and Table 3. 

In 2018 and 2019, 53 faecal samples from sheep were collected from one local slaughterhouse each in Forollhogna (n = 25), Knutshø (n = 18), and Nordfjella (n = 10). The sheep originated from the same farm in each area, and had been grazing on the same natural pastures. The results on prevalence and intensities of parasite eggs and oocysts in faecal samples from domestic sheep are presented in Table 4.

### 3.4. Parasitological Findings in Faecal Samples

Endoparasite eggs or oocysts were detected in 260 (92%) of the 283 wild reindeer faecal samples. A total of 123 (43%) samples contained eggs or oocysts belonging to more than one genus. Strongylida eggs were the most common finding, followed by *N. battus* and *Eimeria* spp. At least 7 genera of parasites were detected altogether.

In sheep samples, endoparasite eggs or oocysts were detected in 52 of 53 samples (98%). A total of 46 (87%) samples contained eggs or oocysts belonging to more than one genus. Strongylida eggs were the most common finding, followed by *Eimeria* spp. and *N. battus*. At least 6 genera of parasites were detected altogether

Parasitological examination of the faecal samples showed that *N. battus* was present in the faeces of reindeer and sheep from all areas, except sheep samples from Forollhogna (Table 2). The prevalence of *N. battus* found in reindeer faecal samples from the different areas were not significantly different when egg counts were compared with a chi-square test (Chi-square = 1.4, df = 2, *p* = 0.50). The prevalence of *N. battus* eggs was higher in reindeer calves (50%) than in adults (1.8%), and the difference was significant when compared with a chi-square test (Chi-square = 84.85, df = 1, *p* < 0.0001). The *N. battus* egg counts from faecal samples from calves in the different areas were significantly different when compared with a Kruskal–Wallis test (H = 12.57, df = 2, *p* = 0.0019), 

### 3.5. Hatching of Nematodirus battus Eggs Isolated from Reindeer Faeces 

Petri dishes with eggs were first incubated at 4 °C for 5–9 weeks, then transferred to an incubator ranging between 11 and 14 °C. After 5 weeks in the 11 and 14 °C incubator, most eggs contained visible live larvae; some eggs were empty with a missing top piece, indicating they had hatched, though no larvae could be observed; and some eggs were whole with shrunken contents and presumed dead. Eggs were screened weekly under a stereomicroscope, and between 5–10 eggs were checked under a light microscope at 100 and 400× magnification by placing one egg on a slide with a drop of water under a coverslip. Eggs with larvae that were left under the microscope hatched after 15–20 min, and the emerging larvae could be observed. After 5–6 weeks of incubation, immature larvae emerged from the eggs, and after 7–10 weeks, the larvae had fully developed into the larger, sheathed infective third stage.

### 3.6. Genetic Diversity of N. battus

**Figure 4:**
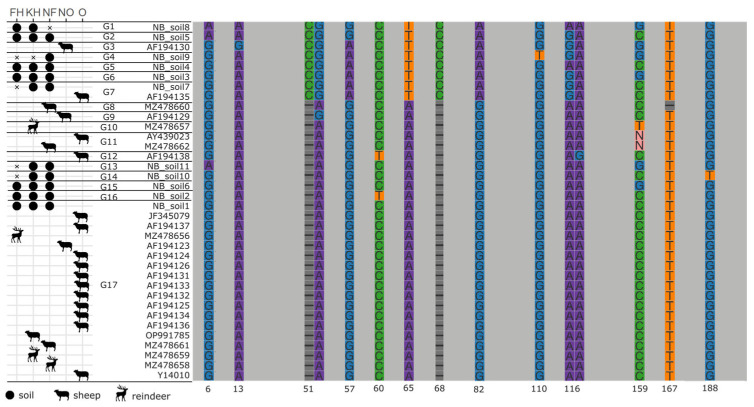
Diagrammatic representation of aligned *Nematodirus battus* ITS2 sequences derived from Sanger sequencing of adult worms and metabarcoding of soil samples. Conserved regions are shown as grey blocks, while variable positions are highlighted in colour with the alignment position indicated below the column. The complete alignment can be found in Appendix A. Horizontal lines between sequence names denote genotype boundaries and the type of sequence (soil, sheep, or reindeer) and geographic provenance are indicated in the table to the left of the diagram. KN: Knutshø; FH: Forollhogna; NF: Nordfjella; NO: Norway; O: other.

The ITS sequences from *N. battus* adult nematodes occurring in sheep, reindeer, and soils in the three management areas were highly similar. A single genotype (G17) was shared among three of four reindeer specimens, two of four sheep specimens, and 97% of soil samples where *N. battus* was detected. This genotype also included sequences from *N. battus* recovered from sheep in Canada, the United States, and the United Kingdom, and other parts of Norway. A single reindeer specimen showed a 1 bp C to T substitution difference from this dominant genotype (Soil D, Appendix A). Fifteen additional *N. battus* ITS2 genotypes were recovered either from soil samples at three or more localities, or from sheep in Norway and other parts of the world (Figure 4, Appendix A).

### 3.7. Metabarcoding of Parasitic Nematodes in Soils

**Figure 5:**
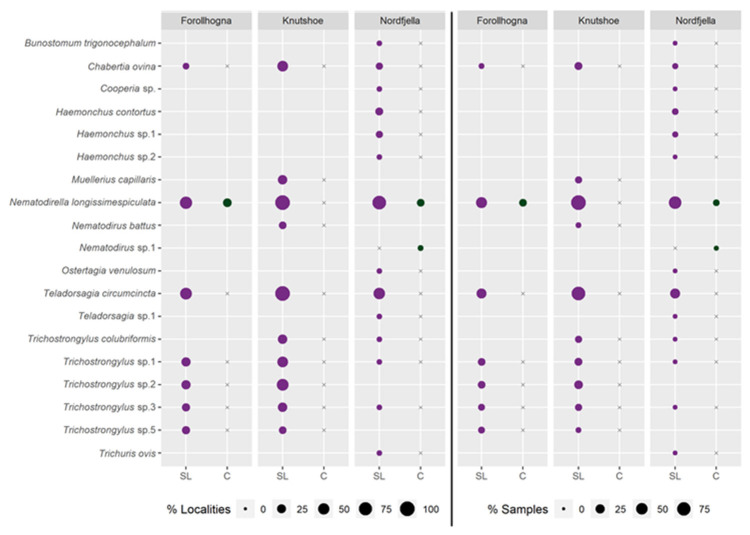
Parasitic nematode DNA detected in active salt lick soil (SL) and controls (C) at different localities in the Forollhogna, Nordfjella, and Knutshø wild reindeer areas. On the left side of the figure, the proportion of positive localities are reported to the left of the centre line, whilst on the right of the figure, the proportion of positive soil samples is reported. Points are sized relative to the percentage of positive localities or samples at which each species was detected. Blanks indicate that a species was absent in the area, while an x indicates the absence of a species in a particular type of location, but a documented presence in the area.

Parasitic nematodes were detected in soil from all the salt lick sites sampled in Knutshø, 80% of sites in Nordfjella, and 69% of sites in Forollhogna. On average, localities in Knutshø harboured a greater diversity of parasitic nematode species per site (mean: 3 [1.1–4.9], range: 1–8) than Forollhogna or Nordfjella (F – mean: 1.9 [0.2–3.6], range: 1–6; N – mean: 1.9 [0.5–3.3], range: 1–6). In all three areas, *N. battus* and *Teladorsagia circumcincta* were the most commonly detected Clade V parasitic nematodes (Figure 5).

Parasitic nematodes were detected significantly more frequently in soil from salt lick sites than in control sites (z = −7.633, *p* << 0.001, Table S3). This pattern was consistent across regions, although the detection of parasitic nematodes was significantly lower at Nordfjella (z = −2.680, *p* = 0.007) than at Forollhogna or Knutshø (Table S2, Figure 5 and Figures S1–S3). The effect of the salt lick site was persistent over time, with abandoned salt lick sites in five localities in Nordfjella and one locality in Forollhogna still exhibiting more frequent detections of parasitic nematodes than surrounding control sites (z = −2.201, *p* = 0.028, Table S4, Figure S4). There was no significant difference in parasitic nematode frequency between abandoned salt lick sites and salt licks in active use at the same locality (z = 0.121, *p* = 0.904), despite 1–4 nematode species being detected at each active salt lick, and only 1–2 nematode species being detected at each abandoned salt lick. 

## 4. Discussion

### 4.1. Evidence for Spillover from Sheep to Reindeer

*Nematodirus battus* was introduced in Norway in 1956, when two rams were imported to southwest Norway from Great Britain [28]. We found *N. battus* eggs in faecal samples from both domestic sheep and wild reindeer in our study, suggesting that spillover of this parasite has occurred between these two hosts. The recovery of *N. battus* with identical ITS genotypes from both sheep and reindeer provides additional support for the spillover hypothesis between domestic and wild animals, as the two species do not appear to harbor unique, host-specific strains of *N. battus.* The ITS region has previously been used to examine genetic variability in *N. battus* across continents, with 11 unique genotypes being previously reported [29]. However, given the moderate variability and relatively short length of the ITS2 region, further analysis using a more robust technique for population genetics such as microsatellites or genotyping by sequencing (GBS) is needed to confidently conclude the two species share the same *N. battus* strain. Nevertheless, the successful development and hatching of *N. battus* eggs isolated from wild reindeer provides additional support that this parasite is able to produce viable eggs when infecting reindeer, which is to our knowledge, a naïve host to *N. battus.* This potentially amplifies the infection potential for all susceptible sympatric animals.

Metabarcoding of soil samples revealed a significantly higher prevalence of parasitic gastrointestinal nematode DNA in salt lick soil compared to soil from control sites. The dominant ITS2 haplotype of *N. battus* recovered from 97% of the soils, where *N. battus* was detected, was identical to that recovered from sheep and wild reindeer faeces. Taken in conjunction with camera trap data, showing intense area use by both sheep and reindeer at salt lick sites, this suggests a major role for salt licks as hot-spots of pathogen transmission.

The nematode species with the highest prevalence in salt lick soils were *N. battus* and *T. circumcincta*. The eggs of these two nematodes are environmentally sturdy and able to survive winters on pasture, which may have led to an accumulation of eggs over time around the frequently visited salt lick sites. 

Since its introduction to Norway, *N. battus* has spread dramatically, and in 2011, it was the predominant *Nematodirus* species identified in Norwegian sheep [30]. Nematodiriosis in lambs caused by *N. battus* can lead to inflammation of the small intestine, causing severe diarrhoea and dehydration, and in severe cases, mortality. Adult sheep normally develop an immune response towards this parasite and are usually not affected [4]. These findings are especially interesting in light of the decrease in wild reindeer calf numbers and slaughter weights previously described. However, the pathological effects of this parasite on reindeer health are, so far, unknown. 

*T. circumcincta* is the most prevalent abomasal nematode species of sheep throughout the world, due to the hardiness of its free-living stages [31]. It is also the most economically important gastrointestinal parasite of sheep in temperate regions [32]. 

Clinical signs of infection are typically dark diarrhoea, dehydration, and reduced weight gain in growing lambs (Type 1 disease). In addition, L4 larvae may re-emerge simultaneously from hypobiosis in late winter, especially during the periparturient relaxation in immunity seen in breeding ewes, causing a subacute or chronic form of teladorsagiosis (Type 2 disease) [33]. In these animals, the immune response to the larvae developing in the abomasal gastric glands impede their ability to digest feed properly [4].

*T. circumcincta* sheds strongyle-type eggs, and are hence indistinguishable from eggs of other trichostrongylid and strongylid species. Consequently, their presence could not be confirmed using morphological examination of helminth eggs found in faecal samples. To explore this, faecal culturing or further molecular studies using metabarcoding or qPCR of faecal samples are warranted, and could provide answers of nematode species diversity as well. It should also be noted that the egg counts presented in this study probably is an underestimation of the true intensity of infection, as only one sample was collected from each animal, and the lag in transport most likely included exposure to higher temperatures in addition to longer than recommended duration from sampling to analysis, which probably led to hatching of strongyle eggs and thus lowering egg counts [34] Nonetheless, as this parasite can survive on frozen and snow-covered pastures for months, its capacity for infecting reindeer has been demonstrated [35], and it is repeatedly detected around salt licks; hence, this parasite is likely already established in the wild reindeer populations of Knutshø, Forollhogna, and Nordfjella. In fact, an experimental study found reindeer to be suitable hosts for ovine infective nematode larvae, amongst other *T. circumcincta* [35]. The parasite went into hypobiosis in the reindeer host in the same manner as in sheep, and may as such be expected to re-emerge in the periparturient period, when the pregnant female is immunosuppressed. 

*T. circumcincta* has been found in both wild and semi-domesticated reindeer in other studies from Norway and Iceland [36,37,38,39], but the impact of *T. circumcincta* infection on reindeer health has not yet been described.

### 4.2. Salt Licks as Transmission Hot-Spots

**Figure 6:**
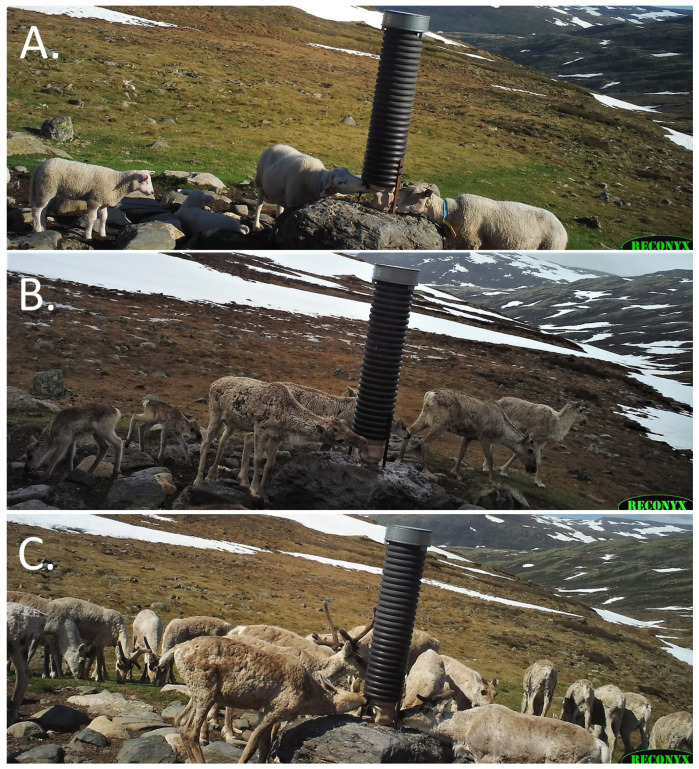
Sample of camera trap data from a single salt lick location in Knutshø during 6–18 June 2020. (**A**). Sheep at a salt lick stone, (**B**). Reindeer calf eating soil, and (**C**). Several reindeer with their nose to the ground “grazing” from the salt lick.

Both natural and artificial hot-spots of animal interaction have been suspected, as well as confirmed as, hot-spots of disease transmission. For example, supplementary feeding was positively associated with the probability of endoparasitic infection in wild boar in a study from Estonia [40]. In a CWD-endemic area in the US, natural mineral licks were found to be hot-spots of transmission due to the accumulation of prions shed from infected animals into soil and water around these licks [41]. However, a study of semi-domesticated reindeer in Finland and Norway did not find supplementary feeding to be a significant factor for infection with endoparasites [42] and no effect of supplementary feeding on gastrointestinal parasite prevalence or infection intensity was found in moose in Norway [43]. Salt lick sites may, however, not be directly comparable with other sites where animals aggregate. In other surroundings, herbivores are expected to avoid faecal contamination [44], but the urge for salt seems to override this. According to our own observations (unpublished), the animals in a reindeer herd typically spread out when they arrive at a salt lick and eagerly lick or eat the salt-laden soil in spite of obvious faecal contamination (Figure 6).

The nematode species with the highest prevalence in salt lick soils were *N. battus* and *T. circumcincta*. There was no significant decline in their prevalence between active and abandoned salt lick sites. The eggs of these two nematodes are environmentally sturdy and able to survive winters on pasture, which may have led to an accumulation of eggs over time around the frequently visited salt licks. Considering the robust nature of these eggs, this could both contribute to increasing infection pressure at salt lick sites used over multiple years, as well as result in a legacy of increased infection pressure around abandoned salt lick sites. However, the current study sampled only a limited number (n = 6) of abandoned salt licks, and as such, additional research is needed to confirm both the magnitude and duration of legacy effects around abandoned salt lick sites, and to quantify infection pressure of parasitic nematodes at these locations.

The collated data on sheep pasture use and reindeer area use suggest that the degree of overlap has increased over the last two decades. In Knutshø, especially, we see that the number of sheep has substantially increased in the early summer pasture areas most frequently used by reindeer females and calves. In this study, we have documented a large number of salt licks within our study areas. There is only anecdotal data on changes in the use of permanent salt licks for sheep, but it may be presumed that the increase in sheep numbers, together with structural changes in sheep production, has led to a proportional increase in number of salt lick sites in areas used by wild reindeer. 

The presence of *N. battus* in reindeer, and the high prevalence of *N. battus* and *T. circumcincta* in salt lick soils, are of particular interest considering the observed decrease in wild reindeer reproduction and calf body mass. Both indices have been shown to be density dependent in ungulate populations, including reindeer [45,46]. Such density dependent effects among ungulates may correlate or even stem from parasitism [47], either through increased direct and indirect (environmental) transmission, or increased susceptibility to parasites due to food shortage and reduced body condition. The three reindeer populations in this study are all regulated by harvest to relatively low densities, with the aim to facilitate high individual body condition and production [48], and the observed long-term reduction in individual reproduction and body mass could not be attributed to a parallel increase in population size (Figure 3). However, during summer, there is a substantial niche overlap between sheep and reindeer [49,50]. Hence, the total number of ungulate grazers has been increasing in reindeer calving/summer areas of Knutshø during the time period, and we cannot exclude grazing intensity as one of the drivers behind the negative trends. 

Even though there were some population fluctuations over the 30-year period, there was no long-term decrease in population sizes following reduced reindeer calf production. This shows that local managers were able to adjust harvest to lower reproduction in the herds. However, the long-term effects on reindeer population dynamics and conservation are yet to be seen. 

The prion disease CWD was found for the first time in Europe in the wild reindeer herd of Nordfjella in 2016 [51], and the herd in Zone 1 (see below) was eradicated during the fall and winter of 2017–2018 to prevent further spread of this disease. Testing of 2359 animals from this subpopulation revealed 19 CWD positive individuals, i.e., a low prevalence [52]. Prions are environmentally resistant pathogens that are excreted with saliva, urine, faeces, etc. and bind to the soil. It has hence been suggested that salt licks may play a role in the CWD epidemiology [41]. However, to study CWD epidemiology in Nordfjella at such a low prevalence would be challenging. In this situation, studies of other environmentally resistant pathogens, such as gastrointestinal nematodes, may provide a proxy that can elucidate the role of salt licks versus the general environment for the transmission of pathogens.

### 4.3. Conclusions

In conclusion, the finding of similar DNA haplotypes of *N. battus* in sheep, reindeer, and in salt lick soil suggests that this sheep parasite has spilled over to reindeer via the salt licks. The high amounts of *N. battus*, *T. circumcincta*, and other gastrointestinal parasites in salt lick soils compared to soils on control sites point to a very important role of the salt licks in the environmental transmission of these pathogens. A similar role may be expected for other environmentally persistent pathogens, like prions. 

These findings, in combination with a concurrent increase in sheep at pasture and presumed increase in the use of the permanent salt licks, as well as the decline in wild reindeer performance, may suggest that increased parasite loads in reindeer due to increased indirect contact with sheep via salt lick soil can have a population impact on wild reindeer. The virulence of the sheep parasites in reindeer and the relative contribution of increased parasite load compared to other factors that may affect reindeer population performance (overgrazing and demographic processes) warrants further investigation. 

## Figures and Tables

**Table 1:** *Nematodirus battus* sequences from adult worms isolated from sheep and reindeer hosts at the three focal locations.

GenBank Accession No.	Management Area	Host
MZ478662.1	Nordfjella	Sheep
MZ478661.1	Nordfjella	Sheep
MZ478660.1	Nordfjella	Sheep
MZ478659.1	Knutshø	Reindeer
MZ478658.1	Knutshø	Reindeer
MZ478657.1	Knutshø	Reindeer
MZ478656.1	Forollhogna	Reindeer
OP991785.1	Knutshø	Sheep

**Table 2:** Helminth eggs and oocysts detected in faecal samples from wild reindeer by geographic area: Forollhogna, Nordfjella, and Knutshø, using McMaster faecal analysis.

Forollhogna (n = 102)	n Positive	% Positive	95% CI	Median epg/opg *	Mean epg/opg	Range of epg/opg
Strongylida	100/102	95.2	91.16–99.31	100	110	20–780
Nematodirinae **	7/102	6.7	1.89–11.44	20	40	10–80
*Nematodirus battus*	17/102	20.2	11.65–28.83	40	32	20–60
*Capillaria* spp.	26/102	24.8	16.51–33.02	25	52	10–300
*Moniezia* spp.	10/102	9.5	4.10–17.33	-	-	-
*Eimeria* spp.	12/102	11.4	5.34–17.51	20	41	20–140
*Trichuris* spp.	1/102	0.9	0–2.8	-	-	80
*Skrjabinema* spp.	6/102	5.7	1.27–10.15	206	222	40–460
**Nordfjella (n = 55)**						
Strongylida	40/55	75.5	63.89–87.06	100	114	10–420
Nematodirinae **	5/55	9.4	1.56–17.30	26	35	20–70
*Nematodirus battus*	7/55	13.2	4.09–22.32	26	29	20–40
*Capillaria* spp.	6/55	11.3	2.79–19.85	20	25	20–40
*Moniezia* spp.	1/55	1.89	0–5.55	-	-	-
*Eimeria* spp.	4/55	7.5	0.44–14.66	160	276	20–764
*Trichuris* spp.	1/55	1.89	0–5.55	-	-	80
*Skrjabinema* spp.	2/55	3.8	0–8.90	350	350	60–640
**Knutshø (n = 126)**						
Strongylida	113/126	91.2	87.04–96.70	150	202	10–1425
Nematodirinae **	10/126	8.1	3.30–12.96	45	85	10–330
*Nematodirus battus*	25/126	20.3	13.21–27.44	50	66	10–300
*Capillaria* spp.	2/126	1.6	0–3.86		43	10–75
*Moniezia* spp.	14/126	11.4	5.77–16.99	-	-	-
*Eimeria* spp.	19/126	15.4	9.06–21.83	75	27,420	20–516,000
*Trichuris* spp.	5/126	5	0.58–7.55	25	43	10–150
*Skrjabinema* spp.	2/126	1.6	0–3.86		360	100–620

** = excluding *N. battus*; * epg = eggs per gram faeces; opg = oocysts per gram faeces.

**Table 3:** Helminth eggs and oocysts detected in faecal samples from wild reindeer by age class: calves and adults, using McMaster analysis of faecal samples.

Calves (n = 79)	n Positive	% Positive	95% CI	Median epg/opg *	Mean epg/opg *	Range of epg/opg *
Strongylida	69	87.3	80.00–94.67	80	168	10–1425
Nematodirinae **	14	17.7	9.3–26.14	45	58	20–180
*Nematodirus battus*	43	54.4	43.45–65.41	40	55	20–310
*Capillaria* spp.	1	1.3	0–3.73		-	20
*Moniezia* spp.	15	18.9	10.34–27.64	-	-	-
*Eimeria* spp.	17	21.5	12.46–30.58	40	30,634	20–51,600
*Trichuris* spp.	2	2.5	0–6.00	23	23	20–25
*Skrjabinema* spp.	7	8.9	2.59–15.13	252	287	40–640
**Adults (n = 149)**						
Strongylida.	133	89.3	84.29–94.23	100	142	20–780
Nematodirinae **	7	4.7	1.30–8.10	10	23	20–26
*Nematodirus battus*	3	2.0	0–4.27	26	51	10–75
*Capillaria* spp.	24	16.1	10.20–22.01	20	42	10–300
*Moniezia* spp.	2	1.3	0–3.19	-	-	-
*Eimeria* spp.	15	10.07	5.24–14.90	75	134	20–764
*Trichuris* spp.	3	2.0	0–4.27	80	115	10–150
*Skrjabinema* spp.	2	1.3	0–3.19	310	310	160–460

* epg = eggs per gram faeces; opg = oocysts per gram faeces; ** = excluding *N. battus*.

**Table 4:** Helminth eggs and oocysts detected in faecal samples from sheep by geographic area: Forollhogna, Nordfjella, and Knutshø, using McMaster faecal analysis.

Forollhogna (n = 25)	n Positive	% Positive	Median epg/opg *	Mean epg/opg *	Range of epg/opg *
Strongylida	20	80	60	70	20–170
*Nematodirus* spp.	2	8	-	60	10–110
*Eimeria* spp.	19	76	400	5547	200–86,200
*Trichuris* spp.	1	4			10
**Nordfjella (n = 10)**					
Strongylida.	10	100	310	351	10–950
*Nematodirus battus*	8	80	185	199	10–460
*Moniezia* spp.	4	40	-	-	-
*Eimeria* spp.	9	90	1700	9250	600–5200
*Trichuris* spp.	5	50			10
**Knutshø (n= 18)**					
Strongylida	18	100	610	652	50–1690
*Nematodirus battus*	13	72.2	60	145	10–580
*Moniezia* spp.	10	55.6	-	-	-
*Eimeria* spp.	16	88.9	1700	9250	200–105,000
*Trichuris* spp.	1	5.6			10

* epg = eggs per gram faeces; opg = oocysts per gram faeces.

## Data Availability

The datasets used and/or analysed during the current study are available from the corresponding author on reasonable request. Statement: The sequence data generated during the current study are available in the NCBI GenBank; accession number PRJNA911771 if not stated elsewhere in this article.

## Supplementary Materials

The following supporting information can be downloaded at: https://www.mdpi.com/article/10.3390/pathogens12020186/s1, Table S1: Overview of soil samples collected from salt lick (SL) and control (C) locations in three management areas during the summer and/or fall of 2017–2019; Table S2: AIC values for models tested and *p*-values for the included variables; Table S3: Odds ratios and confidence intervals for the general linear mixed effects model for parasitic nematode occurrence in soils at active salt licks and control areas in the Forollhogna, Knutshø, and Nordfjella regions; Table S4: Odds ratios and confidence intervals for the general linear model for parasitic nematode occurrence in soils at active and abandoned salt licks and control areas in the Forollhogna and Nordfjella regions; Figure S1: DNA based detection of parasitic nematodes in soils around salt licks and in control areas at 9 localities in Forollhogna; Figure S2: DNA based detection of parasitic nematodes in soils around salt licks and in control areas at 9 localities in Knutshø; Figure S3: DNA based detection of parasitic nematodes in soils around salt licks and in control areas at 26 localities in Nordfjella. Figure S4: DNA based detection of parasitic nematodes in soils around areas previously used as salt licks and in 5 localities in Nordfjella and one in Forollhogna.

## Appendix A

>NB_soil8
ACGAAATACTACAGTATGTCTAGTTATGCTGTATGTCGAATGGTACTATCCGCATAGTCCCGGTTTACCCCATTCAAGTA
AAGAATGTTTGCAACATGGCTTCGTGCTGGTGTCAAAGCCCCTGAATGATGTGGACGCGATTGTAGCCGTATTGAGTTGT
ACTCGATGAGAATGAGATGAATTCAACGGGGAACTGACTGTAGGCAATCGATTGTCCTGACATCATTTGCATTGCAACCT
GAGCTCAGGCGTGACTACCCGCTGAACTTAAGCATATCACTT
>NB_soil5
ACGAAATACTACAGTATGTCTAGTTATGCTGTATGTCGAATGGTACTATCCGCATAGTCCCGGTTTACCCCATTCAAGTA
AAGAATGTTTGCAACATGGCTTCGTGCTGGTGTCAGAGCCCCTGAATGATGTGGACGCGATTGTAGCCGTATTGAGTTCT
ACTCGATGAGAATGAGATGAATTCAACGGGGAACTGACTGTAGGCAATCGATTGTCCTGACATCATTTGCATTGCAACCT
GAGCTCAGGCGTGACTACCCGCTGAACTTAAGCATATCACTT
>AF194130
ACGAAGTACTACGGTATGTCTAGTTATGCTGTATGTCGAATGGTACTATCCGCATAATCCCGGTTTACCCCATTCAAGTA
AAGAATGTTTGCAACATGGCTTCGTGCTGGTGTCAGAGCCCCTGAATGATGTGGACGCGATTGTAGCCGTATTGAGTTGT
ACTCGATGAGAATGAGATGAATTCAACGGGGAACTGACTGTAGGCAATCGATTGTCCTGACATCATTTGCATTGCAACCT
GAGCTCAGGCGTGACTACCCGCTGAACTTAAGCATATCACTT
>NB_soil9
ACGAAGTACTACAGTATGTCTAGTTATGCTGTATGTCGAATGGTACTATCCGCATAATCCCGGTTTACCCCATTCAAGTA
AAGAATGTTTGCAACATGGCTTCGTGCTGTTGTCAGAGCCCCTGAATGATGTGGACGCGATTGTAGCCGTATTGAGTTGT
ACTCGATGAGAATGAGATGAATTCAACGGGGAACTGACTGTAGGCAATCGATTGTCCTGACATCATTTGCATTGCAACCT
GAGCTCAGGCGTGACTACCCGCTGAACTTAAGCATATCACTT
>NB_soil4
ACGAAGTACTACAGTATGTCTAGTTATGCTGTATGTCGAATGGTACTATCCGCATAATCCCGGTTTACCCCATTCAAGTA
AAGAATGTTTGCAACATGGCTTCGTGCTGGTGTCAAAGCCCCTGAATGATGTGGACGCGATTGTAGCCGTATTGAGTTCT
ACTCGATGAGAATGAGATGAATTCAACGGGGAACTGACTGTAGGCAATCGATTGTCCTGACATCATTTGCATTGCAACCT
GAGCTCAGGCGTGACTACCCGCTGAACTTAAGCATATCACTT
>NB_soil3
ACGAAGTACTACAGTATGTCTAGTTATGCTGTATGTCGAATGGTACTATCCGCATAATCCCGGTTTACCCCATTCAAGTA
AAGAATGTTTGCAACATGGCTTCGTGCTGGTGTCAGAGCCCCTGAATGATGTGGACGCGATTGTAGCCGTATTGAGTTGT
ACTCGATGAGAATGAGATGAATTCAACGGGGAACTGACTGTAGGCAATCGATTGTCCTGACATCATTTGCATTGCAACCT
GAGCTCAGGCGTGACTACCCGCTGAACTTAAGCATATCACTT
>NB_soil7
ACGAAGTACTACAGTATGTCTAGTTATGCTGTATGTCGAATGGTACTATCCGCATAATCCCGGTTTACCCCATTCAAGTA
AAGAATGTTTGCAACATGGCTTCGTGCTGGTGTCAGAGCCCCTGAATGATGTGGACGCGATTGTAGCCGTATTGAGTTCT
ACTCGATGAGAATGAGATGAATTCAACGGGGAACTGACTGTAGGCAATCGATTGTCCTGACATCATTTGCATTGCAACCT
GAGCTCAGGCGTGACTACCCGCTGAACTTAAGCATATCACTT
>AF194135
ACGAAGTACTACAGTATGTCTAGTTATGCTGTATGTCGAATGGTACTATCCGCATAATCCCGGTTTACCCCATTCAAGTA
AAGAATGTTTGCAACATGGCTTCGTGCTGGTGTCAGAGCCCCTGAATGATGTGGACGCGATTGTAGCCGTATTGAGTTCT
ACTCGATGAGAATGAGATGAATTCAACGGGGAACTGACTGTAGGCAATCGATTGTCCTGACATCATTTGCATTGCAACCT
GAGCTCAGGCGTGACTACCCGCTGAACTTAAGCATATCACTT
>MZ478660
ACGAAGTACTACAGTATGTCTAGTTATGCTGTATGTCGAATGGTACTATC-ACATAGTCCCGGTATA-CCCATTCAAGTA
AGGAATGTTTGCAACATGGCTTCGTGCTGGTGTCAAAGCCCCTGAATGATGTGGACGCGATTGTAGCCGTATTGAGTTCT
ACTCGA-GAGAATGAGATGAATTCAACGGGGAACTGACTGTAGGCAATCGATTGTCCTGACATCATTTGCATTGCAACCT
GAGCTCAGGCGTGACTACCCGCTGAACTTAAGCATATCACTT
>AF194129
ACGAAGTACTACAGTATGTCTAGTTATGCTGTATGTCGAATGGTACTATC-GCATAGTCCCGGTATA-CCCATTCAAGTA
AGGAATGTTTGCAACATGGCTTCGTGCTGGTGTCAAAGCCCCTGAATGATGTGGACGCGATTGTAGCCGTATTGAGTTCT
ACTCGATGAGAATGAGATGAATTCAACGGGGAACTGACTGTAGGCAATCGATTGTCCTGACATCATTTGCATTGCAACCT
GAGCTCAGGCGTGACTACCCGCTGAACTTAAGCATATCACTT
>MZ478657
ACGAAGTACTACAGTATGTCTAGTTATGCTGTATGTCGAATGGTACTATC-ACATAGTCCCGGTATA-CCCATTCAAGTA
AGGAATGTTTGCAACATGGCTTCGTGCTGGTGTCAAAGCCCCTGAATGATGTGGACGCGATTGTAGCCGTATTGAGTTTT
ACTCGATGAGAATGAGATGAATTCAACGGGGAACTGACTGTAGGCAATCGATTGTCCTGACATCATTTGCATTGCAACCT
GAGCTCAGGCGTGACTACCCGCTGAACTTAAGCATATCACTT
>AY439023
ACGAAGTACTACAGTATGTCTAGTTATGCTGTATGTCGAATGGTACTATC-ACATAGTCCCGGTATA-CCCATTCAAGTA
AGGAATGTTTGCAACATGGCTTCGTGCTGGTGTCAAAGCCCCTGAATGATGTGGACGCGATTGTAGCCGTATTGAGTTNT
ACTCGATGAGAATGAGATGAATTCAACGGGGAACTGACTGTAGGCAATCGATTGTCCTGACATCATTTGCATTGCAACCT
GAGCTCAGGCGTGACTACCCGCTGAACTTAAGCATATCACTT
>MZ478662
ACGAAGTACTACAGTATGTCTAGTTATGCTGTATGTCGAATGGTACTATC-ACATAGTCCCGGTATA-CCCATTCAAGTA
AGGAATGTTTGCAACATGGCTTCGTGCTGGTGTCAAAGCCCCTGAATGATGTGGACGCGATTGTAGCCGTATTGAGTTNT
ACTCGATGAGAATGAGATGAATTCAACGGGGAACTGACTGTAGGCAATCGATTGTCCTGACATCATTTGCATTGCAACCT
GAGCTCAGGCGTGACTACCCGCTGAACTTAAGCATATCACTT
>AF194138
ACGAAGTACTACAGTATGTCTAGTTATGCTGTATGTCGAATGGTACTATC-ACATAGTCTCGGTATA-CCCATTCAAGTA
AGGAATGTTTGCAACATGGCTTCGTGCTGGTGTCAAGGCCCCTGAATGATGTGGACGCGATTGTAGCCGTATTGAGTTCT
ACTCGATGAGAATGAGATGAATTCAACGGGGAACTGACTGTAGGCAATCGATTGTCCTGACATCATTTGCATTGCAACCT
GAGCTCAGGCGTGACTACCCGCTGAACTTAAGCATATCACTT
>NB_soil11
ACGAAATACTACAGTATGTCTAGTTATGCTGTATGTCGAATGGTACTATC-ACATAGTCCCGGTATA-CCCATTCAAGTA
AGGAATGTTTGCAACATGGCTTCGTGCTGGTGTCAAAGCCCCTGAATGATGTGGACGCGATTGTAGCCGTATTGAGTTGT
ACTCGATGAGAATGAGATGAATTCAACGGGGAACTGACTGTAGGCAATCGATTGTCCTGACATCATTTGCATTGCAACCT
GAGCTCAGGCGTGACTACCCGCTGAACTTAAGCATATCACTT
>NB_soil10
ACGAAGTACTACAGTATGTCTAGTTATGCTGTATGTCGAATGGTACTATC-ACATAGTCCCGGTATA-CCCATTCAAGTA
AGGAATGTTTGCAACATGGCTTCGTGCTGGTGTCAAAGCCCCTGAATGATGTGGACGCGATTGTAGCCGTATTGAGTTCT
ACTCGATGAGAATGAGATGAATTCAACTGGGAACTGACTGTAGGCAATCGATTGTCCTGACATCATTTGCATTGCAACCT
GAGCTCAGGCGTGACTACCCGCTGAACTTAAGCATATCACTT
>NB_soil6
ACGAAGTACTACAGTATGTCTAGTTATGCTGTATGTCGAATGGTACTATC-ACATAGTCCCGGTATA-CCCATTCAAGTA
AGGAATGTTTGCAACATGGCTTCGTGCTGGTGTCAAAGCCCCTGAATGATGTGGACGCGATTGTAGCCGTATTGAGTTGT
ACTCGATGAGAATGAGATGAATTCAACGGGGAACTGACTGTAGGCAATCGATTGTCCTGACATCATTTGCATTGCAACCT
GAGCTCAGGCGTGACTACCCGCTGAACTTAAGCATATCACTT
>NB_soil2
ACGAAGTACTACAGTATGTCTAGTTATGCTGTATGTCGAATGGTACTATC-ACATAGTCTCGGTATA-CCCATTCAAGTA
AGGAATGTTTGCAACATGGCTTCGTGCTGGTGTCAAAGCCCCTGAATGATGTGGACGCGATTGTAGCCGTATTGAGTTCT
ACTCGATGAGAATGAGATGAATTCAACGGGGAACTGACTGTAGGCAATCGATTGTCCTGACATCATTTGCATTGCAACCT
GAGCTCAGGCGTGACTACCCGCTGAACTTAAGCATATCACTT
>NB_soil1
ACGAAGTACTACAGTATGTCTAGTTATGCTGTATGTCGAATGGTACTATC-ACATAGTCCCGGTATA-CCCATTCAAGTA
AGGAATGTTTGCAACATGGCTTCGTGCTGGTGTCAAAGCCCCTGAATGATGTGGACGCGATTGTAGCCGTATTGAGTTCT
ACTCGATGAGAATGAGATGAATTCAACGGGGAACTGACTGTAGGCAATCGATTGTCCTGACATCATTTGCATTGCAACCT
GAGCTCAGGCGTGACTACCCGCTGAACTTAAGCATATCACTT
>JF345079
ACGAAGTACTACAGTATGTCTAGTTATGCTGTATGTCGAATGGTACTATC-ACATAGTCCCGGTATA-CCCATTCAAGTA
AGGAATGTTTGCAACATGGCTTCGTGCTGGTGTCAAAGCCCCTGAATGATGTGGACGCGATTGTAGCCGTATTGAGTTCT
ACTCGATGAGAATGAGATGAATTCAACGGGGAACTGACTGTAGGCAATCGATTGTCCTGACATCATTTGCATTGCAACCT
GAGCTCAGGCGTGACTACCCGCTGAACTTAAGCATATCACTT
>AF194137
ACGAAGTACTACAGTATGTCTAGTTATGCTGTATGTCGAATGGTACTATC-ACATAGTCCCGGTATA-CCCATTCAAGTA
AGGAATGTTTGCAACATGGCTTCGTGCTGGTGTCAAAGCCCCTGAATGATGTGGACGCGATTGTAGCCGTATTGAGTTCT
ACTCGATGAGAATGAGATGAATTCAACGGGGAACTGACTGTAGGCAATCGATTGTCCTGACATCATTTGCATTGCAACCT
GAGCTCAGGCGTGACTACCCGCTGAACTTAAGCATATCACTT
>MZ478656
ACGAAGTACTACAGTATGTCTAGTTATGCTGTATGTCGAATGGTACTATC-ACATAGTCCCGGTATA-CCCATTCAAGTA
AGGAATGTTTGCAACATGGCTTCGTGCTGGTGTCAAAGCCCCTGAATGATGTGGACGCGATTGTAGCCGTATTGAGTTCT
ACTCGATGAGAATGAGATGAATTCAACGGGGAACTGACTGTAGGCAATCGATTGTCCTGACATCATTTGCATTGCAACCT
GAGCTCAGGCGTGACTACCCGCTGAACTTAAGCATATCACTT
>AF194123
ACGAAGTACTACAGTATGTCTAGTTATGCTGTATGTCGAATGGTACTATC-ACATAGTCCCGGTATA-CCCATTCAAGTA
AGGAATGTTTGCAACATGGCTTCGTGCTGGTGTCAAAGCCCCTGAATGATGTGGACGCGATTGTAGCCGTATTGAGTTCT
ACTCGATGAGAATGAGATGAATTCAACGGGGAACTGACTGTAGGCAATCGATTGTCCTGACATCATTTGCATTGCAACCT
GAGCTCAGGCGTGACTACCCGCTGAACTTAAGCATATCACTT
>AF194124
ACGAAGTACTACAGTATGTCTAGTTATGCTGTATGTCGAATGGTACTATC-ACATAGTCCCGGTATA-CCCATTCAAGTA
AGGAATGTTTGCAACATGGCTTCGTGCTGGTGTCAAAGCCCCTGAATGATGTGGACGCGATTGTAGCCGTATTGAGTTCT
ACTCGATGAGAATGAGATGAATTCAACGGGGAACTGACTGTAGGCAATCGATTGTCCTGACATCATTTGCATTGCAACCT
GAGCTCAGGCGTGACTACCCGCTGAACTTAAGCATATCACTT
>AF194126
ACGAAGTACTACAGTATGTCTAGTTATGCTGTATGTCGAATGGTACTATC-ACATAGTCCCGGTATA-CCCATTCAAGTA
AGGAATGTTTGCAACATGGCTTCGTGCTGGTGTCAAAGCCCCTGAATGATGTGGACGCGATTGTAGCCGTATTGAGTTCT
ACTCGATGAGAATGAGATGAATTCAACGGGGAACTGACTGTAGGCAATCGATTGTCCTGACATCATTTGCATTGCAACCT
GAGCTCAGGCGTGACTACCCGCTGAACTTAAGCATATCACTT
>AF194131
ACGAAGTACTACAGTATGTCTAGTTATGCTGTATGTCGAATGGTACTATC-ACATAGTCCCGGTATA-CCCATTCAAGTA
AGGAATGTTTGCAACATGGCTTCGTGCTGGTGTCAAAGCCCCTGAATGATGTGGACGCGATTGTAGCCGTATTGAGTTCT
ACTCGATGAGAATGAGATGAATTCAACGGGGAACTGACTGTAGGCAATCGATTGTCCTGACATCATTTGCATTGCAACCT
GAGCTCAGGCGTGACTACCCGCTGAACTTAAGCATATCACTT
>AF194133
ACGAAGTACTACAGTATGTCTAGTTATGCTGTATGTCGAATGGTACTATC-ACATAGTCCCGGTATA-CCCATTCAAGTA
AGGAATGTTTGCAACATGGCTTCGTGCTGGTGTCAAAGCCCCTGAATGATGTGGACGCGATTGTAGCCGTATTGAGTTCT
ACTCGATGAGAATGAGATGAATTCAACGGGGAACTGACTGTAGGCAATCGATTGTCCTGACATCATTTGCATTGCAACCT
GAGCTCAGGCGTGACTACCCGCTGAACTTAAGCATATCACTT
>AF194132
ACGAAGTACTACAGTATGTCTAGTTATGCTGTATGTCGAATGGTACTATC-ACATAGTCCCGGTATA-CCCATTCAAGTA
AGGAATGTTTGCAACATGGCTTCGTGCTGGTGTCAAAGCCCCTGAATGATGTGGACGCGATTGTAGCCGTATTGAGTTCT
ACTCGATGAGAATGAGATGAATTCAACGGGGAACTGACTGTAGGCAATCGATTGTCCTGACATCATTTGCATTGCAACCT
GAGCTCAGGCGTGACTACCCGCTGAACTTAAGCATATCACTT
>AF194125
ACGAAGTACTACAGTATGTCTAGTTATGCTGTATGTCGAATGGTACTATC-ACATAGTCCCGGTATA-CCCATTCAAGTA
AGGAATGTTTGCAACATGGCTTCGTGCTGGTGTCAAAGCCCCTGAATGATGTGGACGCGATTGTAGCCGTATTGAGTTCT
ACTCGATGAGAATGAGATGAATTCAACGGGGAACTGACTGTAGGCAATCGATTGTCCTGACATCATTTGCATTGCAACCT
GAGCTCAGGCGTGACTACCCGCTGAACTTAAGCATATCACTT
>AF194134
ACGAAGTACTACAGTATGTCTAGTTATGCTGTATGTCGAATGGTACTATC-ACATAGTCCCGGTATA-CCCATTCAAGTA
AGGAATGTTTGCAACATGGCTTCGTGCTGGTGTCAAAGCCCCTGAATGATGTGGACGCGATTGTAGCCGTATTGAGTTCT
ACTCGATGAGAATGAGATGAATTCAACGGGGAACTGACTGTAGGCAATCGATTGTCCTGACATCATTTGCATTGCAACCT
GAGCTCAGGCGTGACTACCCGCTGAACTTAAGCATATCACTT
>AF194136
ACGAAGTACTACAGTATGTCTAGTTATGCTGTATGTCGAATGGTACTATC-ACATAGTCCCGGTATA-CCCATTCAAGTA
AGGAATGTTTGCAACATGGCTTCGTGCTGGTGTCAAAGCCCCTGAATGATGTGGACGCGATTGTAGCCGTATTGAGTTCT
ACTCGATGAGAATGAGATGAATTCAACGGGGAACTGACTGTAGGCAATCGATTGTCCTGACATCATTTGCATTGCAACCT
GAGCTCAGGCGTGACTACCCGCTGAACTTAAGCATATCACTT
>OP991785
ACGAAGTACTACAGTATGTCTAGTTATGCTGTATGTCGAATGGTACTATC-ACATAGTCCCGGTATA-CCCATTCAAGTA
AGGAATGTTTGCAACATGGCTTCGTGCTGGTGTCAAAGCCCCTGAATGATGTGGACGCGATTGTAGCCGTATTGAGTTCT
ACTCGATGAGAATGAGATGAATTCAACGGGGAACTGACTGTAGGCAATCGATTGTCCTGACATCATTTGCATTGCAACCT
GAGCTCAGGCGTGACTACCCGCTGAACTTAAGCATATCACTT
>MZ478661
ACGAAGTACTACAGTATGTCTAGTTATGCTGTATGTCGAATGGTACTATC-ACATAGTCCCGGTATA-CCCATTCAAGTA
AGGAATGTTTGCAACATGGCTTCGTGCTGGTGTCAAAGCCCCTGAATGATGTGGACGCGATTGTAGCCGTATTGAGTTCT
ACTCGATGAGAATGAGATGAATTCAACGGGGAACTGACTGTAGGCAATCGATTGTCCTGACATCATTTGCATTGCAACCT
GAGCTCAGGCGTGACTACCCGCTGAACTTAAGCATATCACTT
>MZ478659
ACGAAGTACTACAGTATGTCTAGTTATGCTGTATGTCGAATGGTACTATC-ACATAGTCCCGGTATA-CCCATTCAAGTA
AGGAATGTTTGCAACATGGCTTCGTGCTGGTGTCAAAGCCCCTGAATGATGTGGACGCGATTGTAGCCGTATTGAGTTCT
ACTCGATGAGAATGAGATGAATTCAACGGGGAACTGACTGTAGGCAATCGATTGTCCTGACATCATTTGCATTGCAACCT
GAGCTCAGGCGTGACTACCCGCTGAACTTAAGCATATCACTT
>MZ478658
ACGAAGTACTACAGTATGTCTAGTTATGCTGTATGTCGAATGGTACTATC-ACATAGTCCCGGTATA-CCCATTCAAGTA
AGGAATGTTTGCAACATGGCTTCGTGCTGGTGTCAAAGCCCCTGAATGATGTGGACGCGATTGTAGCCGTATTGAGTTCT
ACTCGATGAGAATGAGATGAATTCAACGGGGAACTGACTGTAGGCAATCGATTGTCCTGACATCATTTGCATTGCAACCT
GAGCTCAGGCGTGACTACCCGCTGAACTTAAGCATATCACTT
>Y14010
ACGAAGTACTACAGTATGTCTAGTTATGCTGTATGTCGAATGGTACTATC-ACATAGTCCCGGTATA-CCCATTCAAGTA
AGGAATGTTTGCAACATGGCTTCGTGCTGGTGTCAAAGCCCCTGAATGATGTGGACGCGATTGTAGCCGTATTGAGTTCT
ACTCGATGAGAATGAGATGAATTCAACGGGGAACTGACTGTAGGCAATCGATTGTCCTGACATCATTTGCAT--------
------------------------------------------.

## References

[1] The Norwegian Biodiversity Information Centre (2021). Results from the 2021 Red List for Species.

[2] Gunn A. (2016). Rangifer tarandus. IUCN Red List Threat. Species.

[3] Solberg E.J., Strand O., Veiberg V., Andersen R., Heim M., Rolandsen C.M., Solem M.I., Holmstrøm F., Jordhøy P., Nilsen E.B. (2017). Cervids 1991–2016: Summary report from The National Monitoring Program for Wild Cervidsvol NINA report 1388. NINA Rep..

[4] Taylor M.A., Coop R.L., Wall R.L. (2016). Parasites of Sheep and Goats (Parasites of the Digestive System). Veterinary Parasitology.

[5] Census of Agriculture. https://www.ssb.no/en/jord-skog-jakt-og-fiskeri/landbrukstellinger/statistikk/landbruksteljing.

[6] Forskrift Om Velferd for Småfe. https://lovdata.no/dokument/SF/forskrift/2005-02-18-160.

[7] Rolandsen C.M., Tveraa T., Gundersen V., Røed K.H., Tømmervik H., Kvie K., Våge J., Skarin A., Strand O. (2022). Klassifisering Av de Ti Nasjonale Villreinområdene Etter Kvalitetsnorm for Villrein. Første Klassifisering-2022.

[8] Pirisinu L., Tran L., Chiappini B., Vanni I., Di Bari M.A., Vaccari G., Vikøren T., Madslien K.I., Våge J., Spraker T. (2018). Novel Type of Chronic Wasting Disease Detected in Moose (*Alces alces*), Norway. Emerg. Infect. Dis..

[9] Beitebruksplan for Folldal. https://www.folldal.kommune.no/_f/p1/i6ce03a2f-c9f7-467b-bdfa-4033d17a0818/beitebruksplan-for-folldal-kommune-2019-2022-vedtatt-av-kommunestyret-140319.pdf.

[10] Beitebruksplan for Rennebu. https://www.rennebu.kommune.no/globalassets/landbruk/vedtattbeiteplan.pdf.

[11] Beiteplan 2009–2012 for Oppdal. https://www.nsg.no/getfile.php/1339525-1352058541/Fylkeslag/S%C3%B8r-Tr%C3%B8ndelag/Dokumenter/Beiteplan%202009-2012%20Oppdal.pdf.

[12] Nilsen E.B., Strand O. (2018). Integrating Data from Multiple Sources for Insights into Demographic Processes: Simulation Studies and Proof of Concept for Hierarchical Change-in-Ratio Models. PLoS ONE.

[13] The RVC/FAO Guide to Veterinary Diagnostic Parasitology. Faecal Examination of Farm Animals for Helminth Parasites. http://www.rvc.ac.uk/review/Parasitology/Index/Index.html.

[14] Davey M.L., Utaaker K.S., Fossøy F. (2021). Characterizing Parasitic Nematode Faunas in Faeces and Soil Using DNA Metabarcoding. Parasit. Vectors.

[15] Pages H., Aboyoun P., Gentleman R., DebRoy S. Biostrings. https://bioconductor.org/packages/Biostrings.

[16] Zhou L., Yu G. Plot Multiple Sequence Alignment Using Ggplot2 R Package Version 1.2.3. http://yulab-smu.top/ggmsa.

[17] Gasser R.B., Chilton N.B., Hoste H., Beveridge I. (1993). Rapid Sequencing of RDNA from Single Worms and Eggs of Parasitic Helminths. Nucleic Acids Res..

[18] Martin M. (2011). Cutadapt Removes Adapter Sequences from High-Throughput Sequencing Reads. EMBnet J..

[19] Callahan B.J., McMurdie P.J., Rosen M.J., Han A.W., Johnson A.J.A., Holmes S.P. (2016). DADA2: High-Resolution Sample Inference from Illumina Amplicon Data. Nat. Methods.

[20] Wang Q., Garrity G.M., Tiedje J.M., Cole J.R. (2007). Naïve Bayesian Classifier for Rapid Assignment of RRNA Sequences into the New Bacterial Taxonomy. Appl. Environ. Microbiol..

[21] Avramenko R.W., Redman E.M., Lewis R., Bichuette M.A., Palmeira B.M., Yazwinski T.A., Gilleard J.S. (2017). The Use of Nemabiome Metabarcoding to Explore Gastro-Intestinal Nematode Species Diversity and Anthelmintic Treatment Effectiveness in Beef Calves. Int. J. Parasitol..

[22] Workentine M.L., Chen R., Zhu S., Gavriliuc S., Shaw N., De Rijke J., Redman E.M., Avramenko R.W., Wit J., Poissant J. (2020). A database for ITS2 sequences from nematodes. BMC Genet..

[23] Nemabiome. http://www.nemabiome.ca.

[24] Bates D., Maechler M., Bolker B., Walker S. (2015). Fitting Linear Mixed-Effects Models Using Lme4. J. Stat. Softw..

[25] Length R. emmeans: Estimated Marginal Means, aka Least-Squares Means. R Package Version 1.8.3. https://CRAN.R-project.org/package=emmeans.

[26] Ripley B.D. (2001). The R Project in Statistical Computing. MSOR Connect.

[27] Lowry R. (2006). VassarStats: Statistical Computation Web Site. Choice.

[28] Helle O. (1969). The Introduction of *Nematodirus battus* (Crofton & Thomas, 1951) into a New Environment. Vet. Rec..

[29] Nadler S.A., Hoberg E.P., Hudspeth D.S.S., Rickard L.G. (2000). Relationships of Nematodirus Species and *Nematodirus battus* Isolates (Nematoda: Trichostrongyloidea) Based on Nuclear Ribosomal DNA Sequences. J. Parasitol..

[30] Domke A.V.M., Chartier C., Gjerde B., Leine N., Vatn S., Stuen S. (2013). Prevalence of Gastrointestinal Helminths, Lungworms and Liver Fluke in Sheep and Goats in Norway. Vet. Parasitol..

[31] Anderson N., Dash K.M., Donald A.D., Southcott W.H., Waller P.J., Donald A.D., Southcott W.H., Dineen J.K. (1978). The Epidemiology and Control of Gastrointestinal Parasites of Sheep in Australia.

[32] Venturina V.M., Gossner A.G., Hopkins J. (2013). The Immunology and Genetics of Resistance of Sheep to *Teladorsagia circumcincta*. Vet. Res. Commun..

[33] Scott P.R. (2015). Sheep Medicine.

[34] Sengupta M.E., Thapa S., Thamsborg S.M., Mejer H. (2016). Effect of Vacuum Packing and Temperature on Survival and Hatching of Strongyle Eggs in Faecal Samples. Vet. Parasitol..

[35] Hrabok J.T., Oksanen A., Nieminen M., Rydzik A., Uggla A., Waller P.J. (2006). Reindeer as Hosts for Nematode Parasites of Sheep and Cattle. Vet. Parasitol..

[36] Bye K. (1987). Abomasal Nematodes from Three Norwegian Wild Reindeer Populations. Can. J. Zool..

[37] Guðmundsdóttir B. (2006). Parasites of Reindeer (Rangifer tarandus) in Iceland. Master’s Thesis.

[38] Robertsen P.A. (2020). Gastrointestinal Parasites in Sympatric Reindeer (*Rangifer tarandus*) and Sheep (*Ovis aries*)-Evidence of Spillover and Consequences Thereof. Master’s Thesis.

[39] Dembereldagva S. (2021). Gastrointestinal Nematodes in Icelandic Reindeer. Master’s Thesis.

[40] Oja R., Velström K., Moks E., Jokelainen P., Lassen B. (2017). How Does Supplementary Feeding Affect Endoparasite Infection in Wild Boar?. Parasitol. Res..

[41] Plummer I.H., Johnson C.J., Chesney A.R., Pedersen J.A., Samuel M.D. (2018). Mineral Licks as Environmental Reservoirs of Chronic Wasting Disease Prions. PLoS ONE.

[42] Jokelainen P., Moroni B., Hoberg E., Oksanen A., Laaksonen S. (2019). Gastrointestinal Parasites in Reindeer (*Rangifer tarandus tarandus*) Calves from Fennoscandia: An Epidemiological Study. Vet. Parasitol..

[43] Milner J.M., Wedul S.J., Laaksonen S., Oksanen A. (2013). Gastrointestinal Nematodes of Moose (*Alces alces*) in Relation to Supplementary Feeding. J. Wildl. Dis..

[44] Gunn A., Irvine R.J. (2003). Subclinical Parasitism and Ruminant Foraging Strategies: A Review. Wildl. Soc. Bull..

[45] Tveraa T., Stien A., Bårdsen B.-J., Fauchald P. (2013). Population Densities, Vegetation Green-up, and Plant Productivity: Impacts on Reproductive Success and Juvenile Body Mass in Reindeer. PLoS ONE.

[46] Albon S.D., Irvine R.J., Halvorsen O., Langvatn R., Loe L.E., Ropstad E., Veiberg V., van der Wal R., Bjørkvoll E.M., Duff E.I. (2017). Contrasting Effects of Summer and Winter Warming on Body Mass Explain Population Dynamics in a Food-Limited Arctic Herbivore. Glob. Chang. Biol..

[47] Bonenfant C., Gaillard J.-M., Coulson T., Festa-Bianchet M., Loison A., Garel M., Loe L.E., Blanchard P., Pettorelli N., Owen-Smith N. (2009). Chapter 5 Empirical Evidence of Density-dependence in Populations of Large Herbivores. Advances in Ecological Research.

[48] Strand O., Nilsen E.B., Solberg E.J., Linnell J.C.D. (2012). Can Management Regulate the Population Size of Wild Reindeer (*Rangifer tarandus*) through Harvest?. Can. J. Zool..

[49] Skogland T. (1984). Wild Reindeer Foraging-Niche Organization. Holarct. Ecol..

[50] Mysterud A. (2000). Diet overlap among ruminants in Fennoscandia. Oecologia.

[51] Benestad S.L., Mitchell G., Simmons M., Ytrehus B., Vikøren T. (2016). First Case of Chronic Wasting Disease in Europe in a Norwegian Free-Ranging Reindeer. Vet. Res..

[52] Mysterud A., Madslien K., Viljugrein H., Vikøren T., Andersen R., Güere M.E., Benestad S.L., Hopp P., Strand O., Ytrehus B. (2019). The Demographic Pattern of Infection with Chronic Wasting Disease in Reindeer at an Early Epidemic Stage. Ecosphere.

