# Supplementary material for: Parasite Spillover from Domestic Sheep to Wild Reindeer—The Role of Salt Licks
Source: Pathogens. 2023 Jan 25;12(2):186. doi: 10.3390/pathogens12020186 (PMC9959430; doi:10.3390/pathogens12020186)

Table S1. Overview of soil samples collected from salt lick (SL) and control (C) locations in three management areas during the summer and/or fall of 2017-2019. Soils were collected both at active salt licks and in areas previously used as salt licks. Grey cells indicate samples for which sequencing failed.

		Old									Active								
		2017	2018				2019				2017	2018				2019			
		Fall	Summer		Fall		Summer		Fall		Fall	Summer		Fall		Summer		Fall	
Area	Locality	SL	SL	C	SL	C	SL	C	SL	C	SL	SL	C	SL	C	SL	C	SL	C
Forollhogna	FH.03	0	0	0	0	0	0	0	0	0	1	1	1	1	1	1	1	1	1
	FH.05	0	0	0	0	0	0	0	0	0	1	1	1	1	1	1	1	0	0
	FH.06	0	0	0	0	0	0	0	0	0	0	0	0	1	1	0	0	0	0
	FH.07	0	0	0	0	0	0	0	0	0	0	1	1	0	0	0	0	0	0
	FH.10	0	0	0	0	0	0	0	0	0	1	0	0	0	0	1	1	0	0
	FH.11	0	0	0	0	0	0	0	0	0	0	1	1	0	0	1	1	1	1
	FH.12	0	0	0	0	0	0	0	0	0	0	1	1	0	0	0	0	0	0
	FH.13	0	0	0	0	0	0	0	0	0	1	0	0	0	0	0	0	0	0
	FH.14	0	0	0	0	0	0	0	0	0	0	1	1	0	0	0	0	0	0
	FH.15	0	0	0	0	0	1	1	0	0	0	0	0	0	0	0	0	0	0
	FH.16	0	0	0	0	0	0	0	0	0	1	0	0	0	0	0	0	0	0
	FH.17	0	0	0	0	0	0	0	0	0	0	0	0	0	0	1	1	0	0
Knutshø	KH.01	0	0	0	0	0	0	0	0	0	0	0	0	1	1	0	0	0	0
	KH.01b	0	0	0	0	0	0	0	0	0	0	0	0	1	1	0	0	0	0
	KH.02	0	0	0	0	0	0	0	0	0	0	1	1	1	1	1	1	1	1
	KH.04	0	0	0	0	0	0	0	0	0	0	1	1	1	1	1	1	1	1
	KH.05	0	0	0	0	0	0	0	0	0	0	1	1	1	1	0	0	0	0
	KH.09	0	0	0	0	0	0	0	0	0	0	1	1	1	1	1	1	0	0
	KH.10	0	0	0	0	0	0	0	0	0	0	1	1	1	1	1	1	1	1
Nordfjella	NF.01	0	0	0	0	0	0	0	0	0	0	0	0	1	1	1	1	0	0
	NF.03b	0	0	0	0	0	0	0	0	0	1	0	0	0	0	0	0	0	0
	NF.04	0	0	0	0	0	0	0	0	0	1	1	1	1	1	1	1	1	1
	NF.05	0	0	0	0	0	0	0	0	0	0	0	0	0	0	1	1	0	0
	NF.06	1	0	0	0	0	0	0	0	0	1	1	1	1	1	0	0	1	1
	NF.07	0	0	0	0	0	0	0	0	0	1	0	0	0	0	0	0	0	0
	NF.08	1	1	1	1	1	1	1	1	1	1	1	1	1	1	1	1	1	1
	NF.09	1	0	0	0	0	0	0	0	0	1	1	1	1	1	0	0	1	1
	NF.10	0	0	0	0	0	0	0	0	0	0	1	1	0	0	1	1	0	0
	NF.11	0	0	0	0	0	0	0	0	0	1	1	1	1	1	0	0	1	1
	NF.12	0	0	0	0	0	0	0	0	0	1	1	1	1	1	0	0	1	1
	NF.13b	1	0	0	0	0	0	0	0	0	1	0	0	0	0	0	0	0	0
	NF.14	0	0	0	0	0	0	0	0	0	0	1	0	1	1	0	0	0	0
	NF.15	0	0	0	0	0	0	0	0	0	1	0	0	0	0	0	0	0	0
	NF.16	0	0	0	0	0	0	0	0	0	0	1	1	0	0	0	0	0	0
	NF.17	0	0	0	0	0	0	0	0	0	0	0	0	0	0	1	1	0	0
	NF.18	0	0	0	0	0	0	0	0	0	0	0	0	0	0	1	1	0	0
	NF.19	0	0	0	0	0	0	0	0	0	0	1	1	1	1	0	0	0	0
	NF.22	0	0	0	0	0	0	0	0	0	0	1	1	0	0	0	0	0	0
	NF.23	0	0	0	0	0	1	1	0	0	0	0	0	0	0	1	1	0	0
	NF.24	0	0	0	0	0	0	0	0	0	0	0	0	0	0	1	1	0	0
	NF.25	0	0	0	0	0	0	0	0	0	0	0	0	1	1	0	0	0	0
	NF.28	0	0	0	0	0	0	0	0	0	0	0	0	0	0	1	1	0	0
	NF.30	0	0	0	0	0	0	0	0	0	0	0	0	0	0	1	1	0	0
	NF.31	0	0	0	0	0	0	0	0	0	0	0	0	0	0	1	1	0	0

Table S2. AIC values for models tested and p-values for the included variables. The selected model presented in the results indicated in bold and variables not included in the model are shaded grey. The intercept from which contrasts are calculated in all models is the control samples from Forollhogna. KN: Knutshø, NF:Nordfjella.

Model	AIC	Salt Lick	AreaKN	AreaNF
Null Model	515.1			
Salt Lick	373.9	<0.001		
Area	507.8		<0.001	<0.001
**Salt Lick+Area**	**365.1**	**<0.001**	**0.492**	**0.007**

Table S3. Odds ratios and confidence intervals for the general linear mixed effects model for parasitic nematode occurrence in soils at active salt licks and control areas in the Forollhogna, Knutshø, and Nordfjella regions. FH: Forollhogna, KN: Knutshø, NF: Nordfjella, SE: Standard Error, LCL: Asymptotic Lower Confidence Limit, UCL: Asymptotic Upper Confidence Limit

**Contrast**	**Odds Ratio**	**SE**	**LCL**	**UCL**
**Salt Lick:Control**	25.301	10.710	11.037	58.003
**FH/KN**	0.737	0.327	0.260	2.087
**FH/NF**	2.699	1.000	1.133	6.431
**KN/NF**	3.662	1.381	1.513	8.860

Table S4. Odds ratios and confidence intervals for the general linear model for parasitic nematod occurrence in soils at active and abandoned salt licks and control areas in the Forollhogna and Nordfjella regions. AB: Abandoned salt lick; AC: Active salt lick; CON: Control; SE: Standard Error, LCL: Asymptotic Lower Confidence Limit, UCL: Asymptotic Upper Confidence Limit

**Contrast**	**Odds Ratio**	**SE**	**LCL**	**UCL**
**AB/AC**	0.941	0.473	0.290	3.055
**AB/CON**	10.487	11.196	0.859	128.042
**AC/CON**	11.143	11.821	0.927	133.911

Figure S1. DNA based detection of parasitic nematodes in soils around salt licks and in control areas at 9 localities in Forollhogna. Coloured circles indicate detection, x indicates an absence, while blanks indicate no samples were collected. The bar chart to the right shows the number of samples collected of each type at each location.

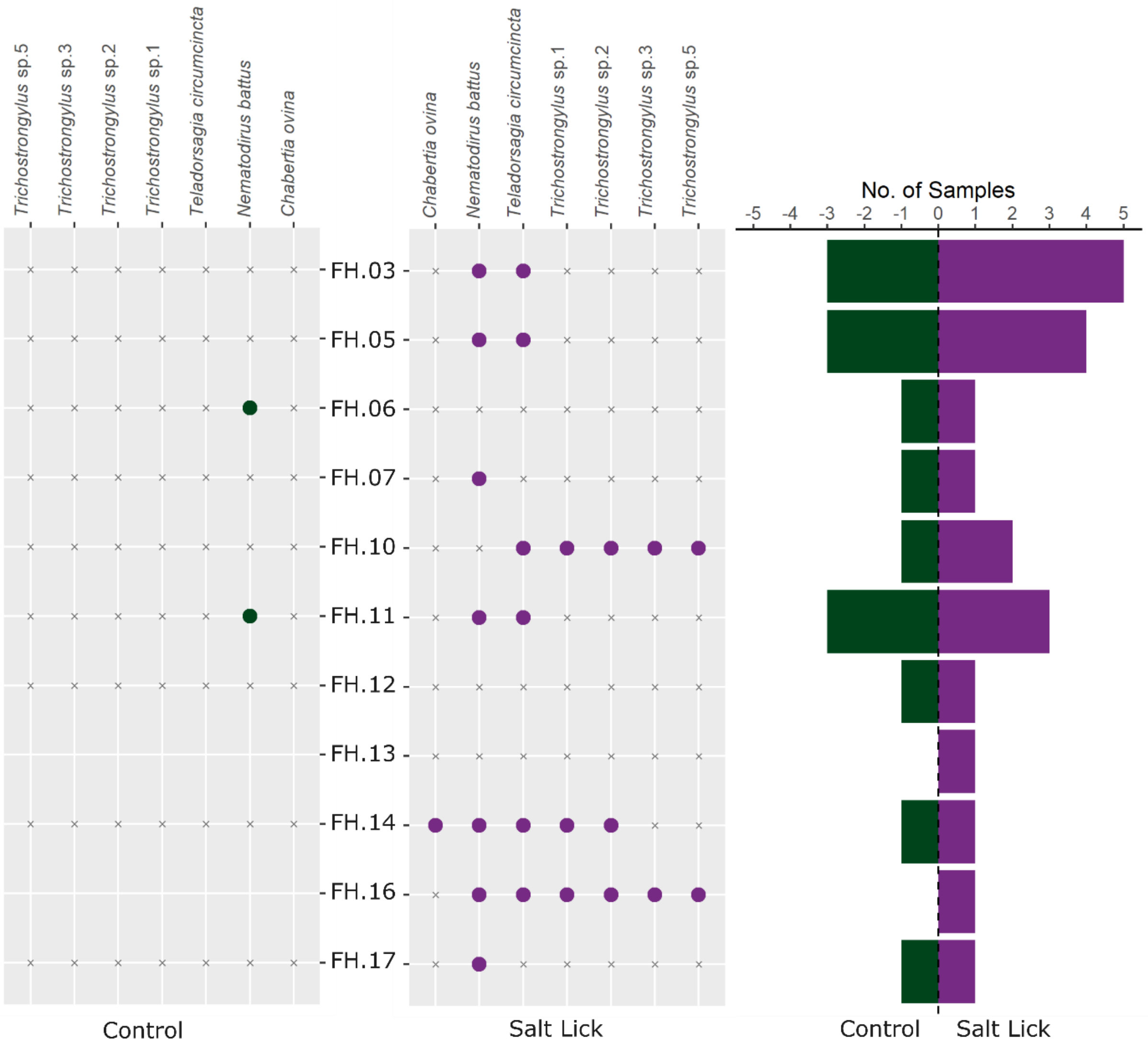

Figure S2. DNA based detection of parasitic nematodes in soils around salt licks and in control areas at 9 localities in Knutshø. Coloured circles indicate detection, x indicates an absence, while blanks indicate no samples were collected. The bar chart to the right shows the number of samples collected of each type at each location.

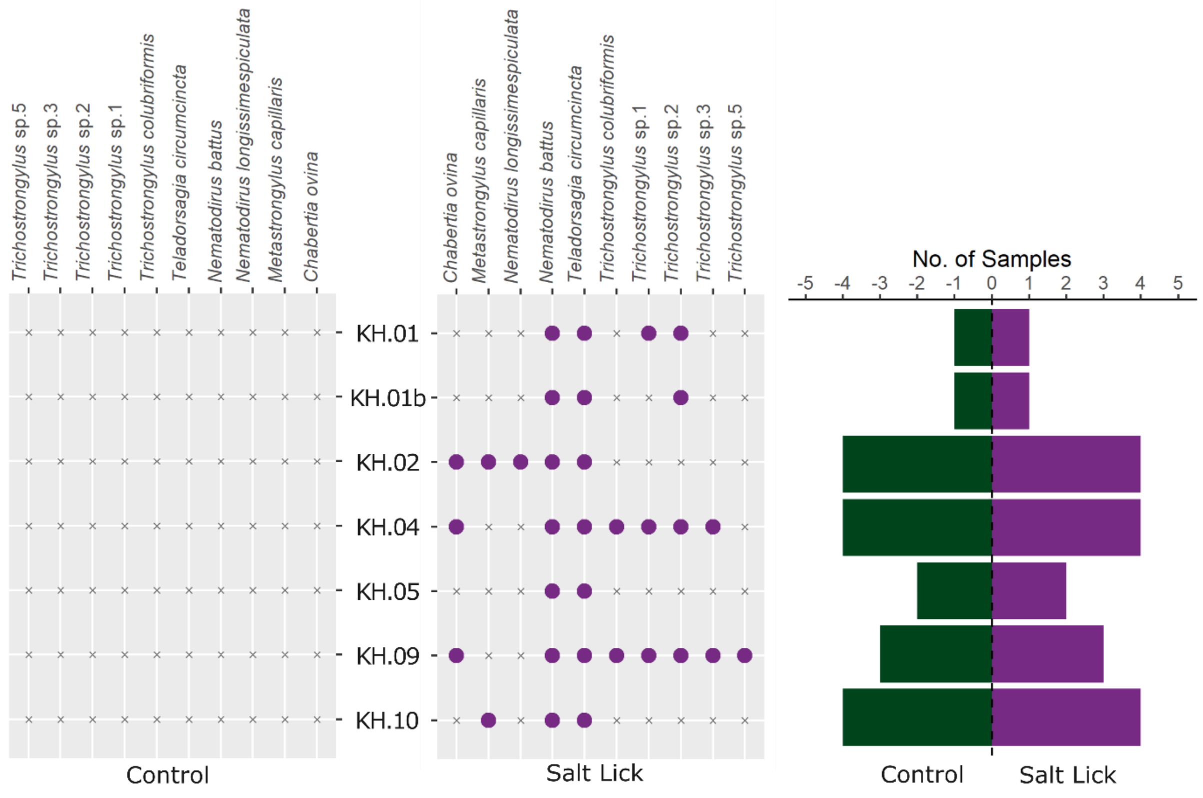

Figure S3. DNA based detection of parasitic nematodes in soils around salt licks and in control areas at 26 localities in Nordfjella. Coloured circles indicate detection, x indicates an absence, while blanks indicate no samples were collected. The bar chart to the right shows the number of samples collected of each type at each location.

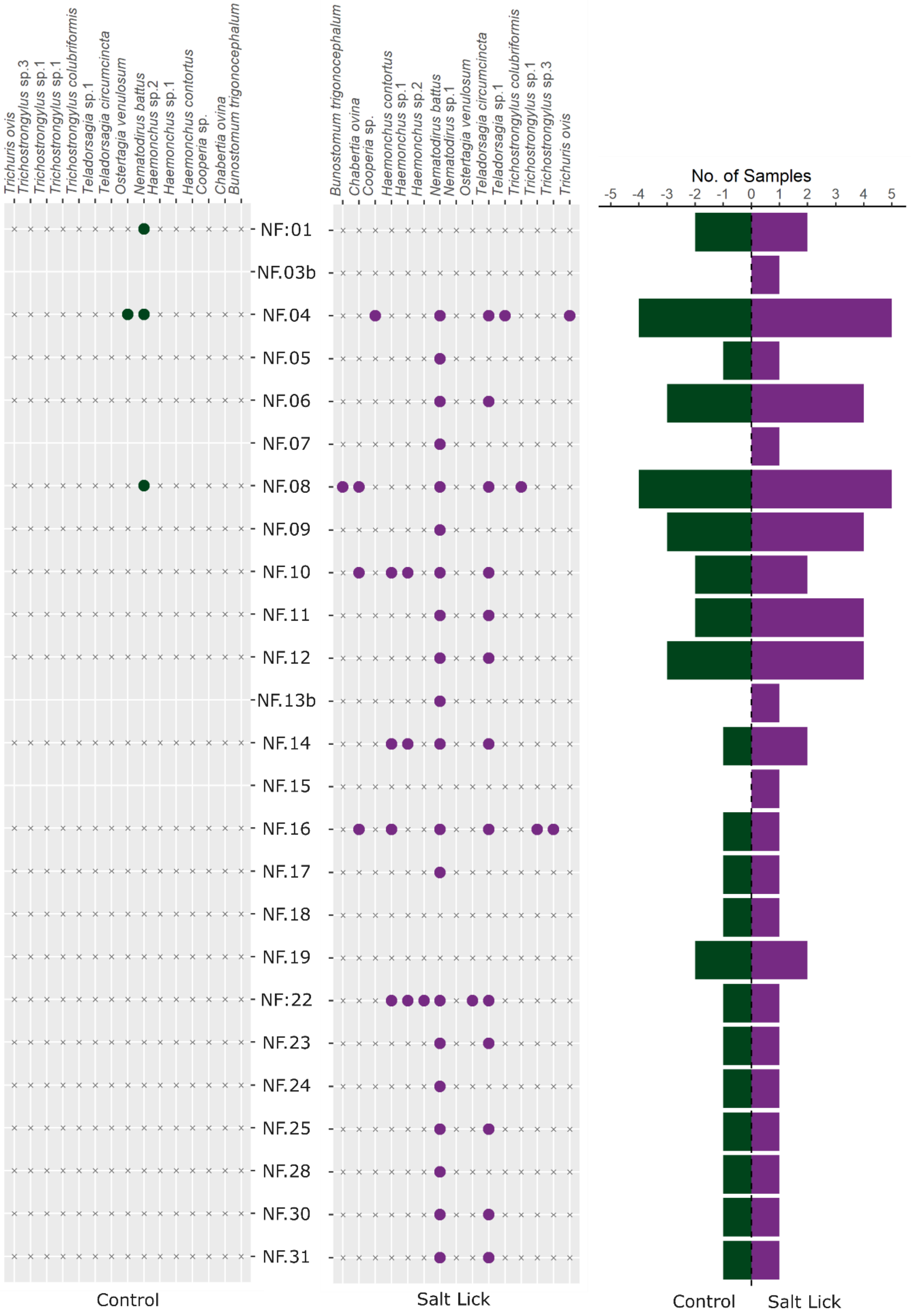

Figure S4. DNA based detection of parasitic nematodes in soils around areas previously used as salt licks and in 5 localities in Nordfjella and one in Forollhogna. Coloured circles and stars indicate detection in active and abandoned salt licks, respectively, while x indicates an absence. Each column represents samples from a single location:sampling campaign combination. AC: Active Control; ASL: Active Salt Lick; OC: Abandoned Control; OSL: Abandoned Salt Lick.

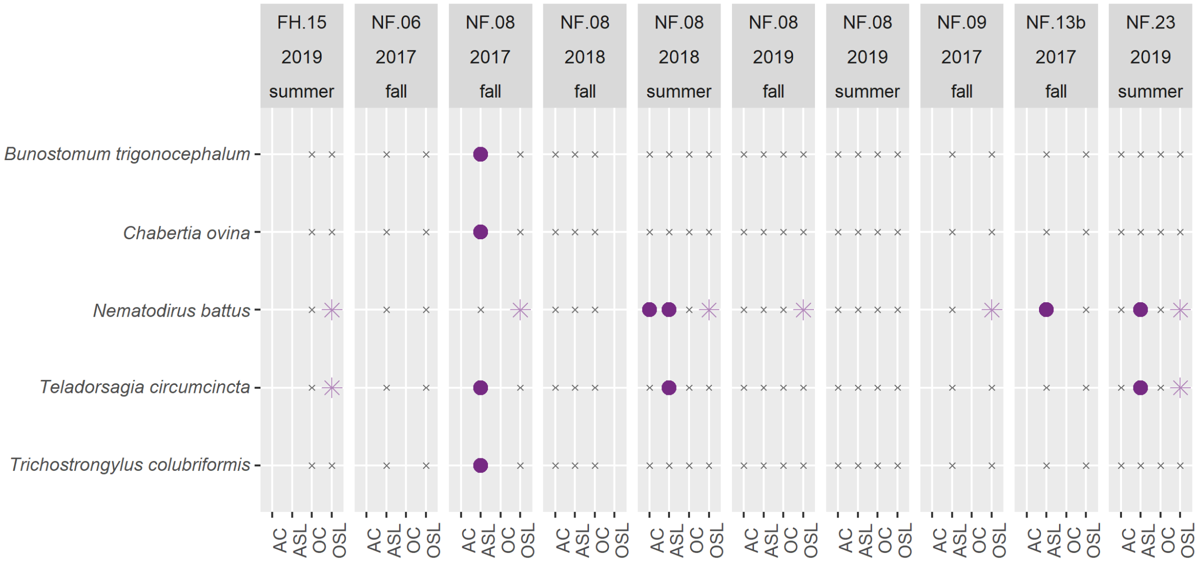

Supplement: Supplementary file 1 [file pathogens-12-00186-s001.zip › pathogens-1996417-supplementary.pdf]
